# Oral Delivery of R‐spondin1‐Loaded Small Extracellular Vesicles Activates WNT Signalling Pathway to Accelerate Intestinal Injury Repair and Reverse Ageing

**DOI:** 10.1002/jev2.70226

**Published:** 2026-01-22

**Authors:** Lingyan Yang, Xu Wang, Xiyang Wei, Pei Yu, Yue Liu, Shixiang Wang, Yuefang Lin, Yue Yang, Ting Jiang, Zhiping Qiao, Jiaxiang Zhang, Shicheng Yu, Ye‐Guang Chen, Yun‐Shen Chan

**Affiliations:** ^1^ Guangzhou National Laboratory Guangzhou Guangdong Province China; ^2^ The Fifth Affiliated Hospital Guangzhou Medical University Guangzhou Guangdong Province China; ^3^ The Third Affiliated Hospital Guangzhou Medical University Guangzhou Guangdong Province China; ^4^ Zhongshan School of Medicine Sun Yat‐Sen University Guangzhou Guangdong Province China; ^5^ State Key Laboratory of Medicinal Chemical Biology, College of Life Sciences Nankai University Tianjin China; ^6^ The MOE Basic Research and Innovation Center for the Targeted Therapeutics of Solid Tumors, Institute of Organoid Research, Jiangxi Medical College Nanchang University Nanchang Jiangxi Province China; ^7^ The State Key Laboratory of Membrane Biology, Tsinghua‐Peking Center for Life Science Tsinghua University Beijing China

**Keywords:** ageing reversal, extracellular vesicles, intestinal regeneration, R‐spondin1, WNT signalling

## Abstract

The intestine plays a crucial role in regulating metabolism and immunity, with functional decline occurring during injury and ageing. Stimulating the neogenesis of intestinal stem cells (ISCs) by activating the WNT/β‐catenin signalling pathway represents a promising approach for intestinal tissue regeneration and injury repair. However, effective oral delivery of functional WNT signalling agonists to the gut remains challenging. Herein, we report a potent WNT/β‐catenin signalling‐inducing small extracellular vesicles (sEV) that can be administered orally and present remarkable therapeutic efficacy. We demonstrate that active R‐spondin1 (RSPO1) protein can be loaded onto the surface of sEV via heparan sulfate proteoglycans. Notably, sEV‐delivered RSPO1 (evRSPO1) effectively induces WNT/β‐catenin signalling‐inducing activity, enhances ISCs proliferation, and supports intestinal organoid growth in vitro. Importantly, oral administration of evRSPO1 activates the WNT/β‐catenin signalling pathway in the cryptic stem cell niche, thereby accelerating tissue repair and regeneration in a radiation‐induced intestinal injury model. Furthermore, evRSPO1 treatment induces ISCs proliferation and reverses the intestinal senescence phenotype in aged mice. Collectively, this study establishes evRSPO1 as a potential first‐in‐class, orally deliverable therapeutic that overcomes biological barriers to activate ISCs, enabling efficient intestinal tissue repair and rejuvenation.

## Graphical abstract

Yang et al. demonstrated that HSPG mediate the loading of active R‐spondin1 protein onto the surface of small extracellular vesicles (evRSPO1). evRSPO1 induced WNT/β‐catenin activity in intestinal organoids and tissues, thereby regulating intestinal stem cells proliferation. Oral administration of evRSPO1 accelerated tissue regeneration in radiation‐induced intestinal injury and reversed senescence phenotype in mice.

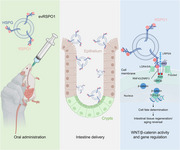

## 1 Introduction

The intestine serves as the body's largest endocrine and immune interface, orchestrating nutrient absorption while maintaining barrier integrity against pathogens (Bany Bakar et al. 2023). Acute injury disrupts mucosal homeostasis and impairs the intestinal epithelial barrier (Barker et al. 2007). In response, intestinal stem cells (ISCs) initiate intestinal epithelial regeneration through proliferation and differentiation, preserving intestinal integrity (Choi and Augenlicht, 2024). This process is regulated by multiple important signalling pathways (Liu et al. 2024; Stojanović et al. 2022). Among these, the canonical WNT/β‐catenin signalling pathway plays a pivotal role in sustaining the self‐renewal and proliferation of ISCs (Yan et al. 2017). ISCs in the crypts, marked by *Lgr5* expression, a major downstream target of WNT/β‐catenin signalling, are the dynamic source of intestinal epithelial cells for self‐renewal (Sato et al. 2011; Snippert et al. 2010).

When extracellular WNT ligands bind to their Frizzled receptor and co‐receptors, LRP5/6, they recruit the intracellular degradation complex and release β‐catenin, which then accumulates in the cytoplasm and translocates to the nucleus, and drives transcription of WNT target genes (Clevers and Nusse, 2012; Perugorria et al. 2019). However, WNT/β‐catenin signalling is rapidly attenuated by transmembrane RING finger ubiquitin ligases Zinc and RING finger 3 (Znrf3) and RING finger 43 (Rnf43), which degrade the Frizzled and LRP5/6 receptors (Farnhammer et al. 2023). The R‐spondin (RSPO) family enhances WNT/β‐catenin signalling by binding to their specific receptors, the leucine‐rich repeat‐containing G‐protein‐coupled receptors (LGR4, 5, and 6), and preventing the Znrf3/Rnf43‐mediated degradation of Frizzled and LRP receptors, thereby stabilizing these receptors and potentiating WNT signalling (de Lau et al. 2011). RSPO1 exerts potent mitogenic effects on WNT‐dependent adult ISCs both in vivo (Kim et al. 2005) and in vitro (Sato et al. 2009). It promotes crypt cell expansion and mucosal repair in DSS‐ and TNBS‐induced colitis models (Zhao et al. 2007). A subset of the intestinal stromal cells serves as the primary cellular source of RSPO1 following intestinal injury in mice (Wu et al. 2021). Consequently, RSPO1 is recognized as a major driver of WNT‐dependent crypt self‐renewal with therapeutic potential for gastrointestinal disorders.

Despite its therapeutic potential, several translational challenges hinder the clinical applications of RSPO1. RSPO1 tends to form dimers or multimers during purification, which may reduce their binding affinity to LGR4/5 receptors and thereby directly compromise the potentiation of WNT/β‐catenin signalling (Kim et al. 2008; Zebisch et al. 2013). Dimeric proteins often have more complex folding and assembly requirements, which can complicate large‐scale production and long‐term stability. The larger size of the dimer may affect tissue penetration, absorption, and in vivo half‐life, potentially limiting bioavailability. Multimeric protein complexes can increase the risk of immune recognition and neutralizing antibody formation (Carmon et al. 2011; Hao et al. 2016). Additionally, natural RSPO1 contains multiple glycosylation modification sites, which are crucial for maintaining its stability and activity and may be lost during protein purification (Chang et al. 2016; Tsuchiya et al. 2016). Moreover, storage conditions such as temperature, pH, and repeated freeze‐thaw cycles may lead to protein degradation or aggregation, further reducing its activity (Levin et al. 2020). Consequently, RSPO1 conditional medium is commonly used to activate WNT signalling and support the growth of stem cells and organoids in vitro (Heuberger et al. 2025; Qu et al. 2021). However, such conditioned media contain undefined additives or serum components, which confound experimental outcomes, limit reproducibility, and preclude therapeutic development.

Small extracellular vesicles (sEV) offer a solution to address the limitations of protein delivery. These naturally secreted nano‐sized extracellular vesicles facilitate intercellular communication via diverse cargoes, including proteins, nucleic acids, and lipids (Fusco et al. 2024; Huang et al. 2023). The cargoes are protected from degeneration by the lipid bilayer membrane, indicating that sEV may serve as an alternative natural delivery vehicle. In addition to being internalized via endocytosis or membrane fusion, sEV surface proteins can directly engage recipient cell receptors, eliciting effects independent of cargo transfer (Cerrotti et al. 2024).

In this study, we show that sEV can actively carry RSPO1 (evRSPO1) on their membrane surface via heparan sulfate proteoglycans (HSPGs). evRSPO1 significantly induces high WNT/β‐catenin pathway activity in vitro and in vivo, promoting crypt growth in intestinal organoids more effectively than human recombinant RSPO1 protein (hrRSPO1). Furthermore, oral administration of evRSPO1 enhances ISCs replication and accelerates intestinal regeneration in radiation‐induced injury and reverses the senescence phenotypes in intestinal tissue of aged mice. These results demonstrate a previously unexplored strategy for delivering RSPO1 via sEV and suggest a potential approach for treating intestinal diseases through tissue regeneration.

## 2 Materials and Methods

### 2.1 Animal Studies

C57BL/6J mice (wild‐type, male, 6∼8 weeks for injury model or 18 months for ageing experiment) were purchased from Gempharmatech (Guangdong, China). Axin2‐mGFP mice were kindly provided by Dr. Yi Arial Zeng, and Lgr5‐EGFP mice were maintained in the lab of Prof. Ye‐Guang Chen. All experimental procedures were approved by the Ethics Committee of Guangzhou National Laboratory (AUCP‐2022‐12‐A02) and performed under the Guide for the Care and Use of Laboratory Animals.

### 2.2 sEV Isolation and Characterization

The plasmid for human RSPO1 protein expression was constructed. Briefly, Human RSPO1 fused to a C‐terminal 6×His tag was synthesized by Igebio (Guangzhou, China) and subcloned into the pLVX‐MSC‐IRES‐PuroR plasmid via the EcoRI and XbaI restriction sites under the control of the EF1α promoter. The integrity of the inserted sequences was confirmed by Sanger sequencing using the primer EF1A‐F (5′‐CTGGGAAAGTGATGTCGTGT‐3′). HEK293 cells and HEK293T cells were cultured in Dulbecco's modified Eagle's medium (DMEM) (Cytiva, HyClone, USA) supplemented with 10% fetal bovine serum (Gibco, USA) and 1% penicillin/streptomycin (Gibco, USA) at 37°C in a humidified atmosphere containing 5% CO_2_ in an incubator (Thermo, USA). Then, HEK293T cells were transfected with the RSPO1‐IRES‐Puromycin plasmid and the co‐transfection plasmid, and the lentiviruses were harvested at 24 and 48 h. HEK293 cells were infected with the lentiviruses for 48 h and selected with puromycin (Gibco, USA). Based on the TOPFlash luciferase activity of the culture supernatant, the monoclonal cell was selected. After expansion culture, the cells were cultured in a serum‐free medium (Yocon, China) on a shaker at 100 rpm. When the cell density reached 10^7^ cells/mL, as detected by the automatic cell counter (EVE PLUS, NanoEntek, Korea), the cells were separated with 170 g for 3 min, and then the supernatant was removed and the debris with 2000 g for 10 min. After filtering with a 0.22 µm filter to remove the large vesicles, the supernatant was used to isolate sEV by EXODUS (H600, Huixin, China). The morphology, size distribution, and concentration of sEV were characterized by TEM (Talos L120C, Thermo, USA) and Flow Nano Analyzer (Nanofcm, China).

### 2.3 TOPFlash Luciferase Activity Report Assay

The Firefly TOPFlash luciferase report plasmid and Renilla luciferase plasmid were constructed. Briefly, the TOPFlash reporter plasmid was constructed by inserting eight tandem TCF/LEF response elements (5′‐A/T A/T CAAAG‐3′) and a minimal promoter upstream of the firefly luciferase gene. The Renilla control plasmid was designed to constitutively express Renilla luciferase under the control of the SV40 promoter. HEK293T cells were cultured in a 96‐well plate overnight and transfected with both plasmids with lipo8000 (Beyotime, China) for 24 h and then treated with sEV or RSPO1 protein, or no treatment (untreated) for another 24 h. After treatment, cell lysates were prepared and analysed using a Dual‐Luciferase Report Assay System according to the manufacturer's instructions (Promega, USA). The absorbance was determined at 560 nm, the Renilla luciferase was included as a transfection control, and the relative fold change was obtained after normalization with the untreated group.

### 2.4 Immunogold Labelling

An immunogold labelling experiment was performed by referring to a previous report (Zhang et al. 2022). sEV were resuspended with an equal volume of 4% paraformaldehyde, then placed onto a 400‐mesh carbon/formvar coated grid and allowed to absorb for 20 min. The grids were blocked with 5% BSA (w/v) in PBS for 10 min and incubated with the primary anti‐RSPO1 antibodies (R&D Systems, USA) for 3 h at room temperature. Then, the grids were incubated with a secondary anti‐mouse IgG antibody (Sigma, USA) conjugated to 10‐nm gold particles (Merck, Germany) for 1 h, washed with PBS, and placed in 1% glutaraldehyde (w/v) in PBS for 5 min. After rinsing in PBS, the grids were stained with uranyl acetate and observed using a transmission electron microscope.

### 2.5 Abbelight STORM

sEV were captured on CD9/CD63/CD81 antibody‐coated coverslips to isolate single vesicles, labelled with Alexa Fluor 647‐conjugated anti‐CD63 antibodies (Abbelight, France), along with antibodies against RSPO1 (R&D Systems, USA), which were then labelled with Alexa Fluor 488. Sequential excitation with 640 and 488 nm lasers was performed, and 10,000 frames per ROI were acquired at 20 ms exposure. Auto‐focus lock minimized z‐drift during imaging. Data were processed with Abbelight NEO software for super‐resolution reconstruction and drift correction. Imaging was carried out on an Abbelight SAFe 180 STORM system mounted on an Evident IX83 inverted microscope with a 100× 1.49 NA TIRF objective.

### 2.6 Heparinase I/III Treatment

Heparinase I and III Blend (Sigma, Germany) reconstituted in 20 mM Tris‐HCl (pH 7.5) with 0.1 mg/mL BSA and 4 mM CaCl_2_. 1 × 10^11^ particles/mL evRSPO1 were incubated with 50 U/mL Heparinase I/III, 5:1 mixture, in a final volume of 100 µL for 1.5 h at 37°C on a shaker. Control sEV were incubated with the reconstitution solution alone. Samples were purified again and detected by Western blot and Luciferase assay.

### 2.7 Human RSPO1 Protein Enzyme‐Linked Immunosorbent Assay

RSPO1 protein loading efficiency on the sEV was measured with an enzyme‐linked immunosorbent assay (ELISA) kit (R&D Systems, USA). Briefly, a 96‐well microplate was pre‐coated with capture antibody and incubated overnight at 4°C. The coating antibody was aspirated, and the plate was rinsed with washing buffer. After blocking with 1% BSA, sEV and hrRSPO1 protein samples or standards were incubated for 1.5 h at room temperature. After removing the samples, the protein‐specific antibody was added and incubated for 1.5 h at room temperature. The antibody was removed, and samples were rinsed with the washing buffer three times. Subsequently, streptavidin‐HRP was added and incubated for 20 min at room temperature. After aspiration and washing, the TMB substrate was added to each well and incubated for 5 min before adding the stop solution. The optical density was measured using a microplate reader set to 450 nm.

### 2.8 Liquid Chromatography‐Tandem Mass Spectrometry Analysis

The sEV samples were reduced with 10 mM dithiothreitol for 30 min at room temperature and then alkylated with 20 mM iodoacetamide for 30 min at room temperature in the dark. Subsequently, the protein mixtures were incubated with Lys‐C (enzyme/protein, 1:100, w/w) for 4 h and trypsin (enzyme/protein, 1:50, w/w) for 12 h at 37°C. Peptide samples were analysed by LC‐MS/MS by combining a Vanquish Neo UHPLC connected online to an Orbitrap Astral mass spectrometer (Thermo, USA). The raw data were processed by Spectronaut v19 (Biognosys) with the ‘Direct‐DIA’ mode for protein identification and quantification. All data were searched against a human database downloaded from UniProt.

### 2.9 Intestinal Organoid Culture and sEV Treatment

Intestinal crypts were isolated from Axin2‐mGFP reporter mice (young and aged) following established methodologies with modifications (Wei, Yu, Zhang, et al. 2023). Briefly, mice were euthanized by cervical dislocation, and the small intestine was excised from the gastroduodenal junction to the ileocecal valve. Luminal contents were flushed with ice‐cold PBS, and the intestine was longitudinally opened and sectioned into 5–10 mm fragments. Subsequent removal of villi was achieved by incubating fragments in 2 mM EDTA/PBS at 4°C for 30 min with gentle agitation, followed by mechanical dissociation via luminal surface scraping to preserve crypt integrity. Tissue fragments were vigorously vortexed in cold PBS, and the crypt‐enriched suspension was filtered through a 70‐µm cell strainer (BD Biosciences, USA). Crypts were pelleted by centrifugation (300 g, 3 min) and resuspended in 0.1% BSA for quantification. For organoid culture, crypts were resuspended in growth factor‐reduced Matrigel (Corning, USA) at a density of 200–500 crypts/20 µL and plated as domes in 24‐well plates. Matrigel was polymerized at 37°C for 10 min, followed by the addition of complete medium consisting of Advanced DMEM/F12 (Gibco, USA), 50 ng/mL EGF (Novoprotein, China), 100 ng/mL Noggin (R&D Systems, USA), 500 ng/mL RSPO1 (R&D Systems, USA), 1× N_2_ (Gibco, USA), 1× B_27_ (Gibco, USA), 1× GlutaMAX (Gibco, USA), 1 mM N‐acetylcysteine (Sigma, Germany) and 1% penicillin/streptomycin. Medium was replenished every 3 days. For passaging, organoids were mechanically dissociated in cold PBS, pelleted (300 × g, 3 min), and re‐embedded in fresh Matrigel every 7 days. For sEV treatment, organoids were dissociated into single cells and embedded in Matrigel. sEV were diluted in medium without RSPO1 protein to concentrations standardized by particle count (∼1 × 10^9^ particles/mL, ∼100 ng/mL RSPO1 protein), and added to culture for 48 h. The negative control included medium without any hrRSPO1 protein, and the positive control included medium with 100 ng/mL hrRSPO1 protein. For quantification of buddings on the organoids, only organoids with a diameter greater than 200 µm were selected in each field of view, and the average was calculated; there are a total of three fields of view.

### 2.10 sEV Cellular Uptake and In Vivo Distribution With Oral Administration

sEV were stained with 10 µM PKH26 (Sigma, Germany) for 20 min at room temperature, and the residual dye was removed by purification again. 500 µL of PKH26‐labelled sEV (1 × 10^9^ particles/mL) for a 24‐well plate was incubated with intestinal organoids for 12 h. After fixing with 4% PFA and staining with FITC‐Phalloidin (Beyotime, China) and DAPI (Sigma, Germany), sEV cellular uptake was observed using confocal microscopy. To determine the in vivo distributions, sEV were stained with 10 µM PKH26 or 10 µM DiR (Meilun Bio, China) for 20 min at room temperature, and the residual dye was removed. 200 µL labelled sEV (∼2 × 10^10^ particles) were treated by oral administration. The biodistribution images were captured using the IVIS Lumina system (PerkinElmer, USA), and the tissue frozen section images were obtained using the confocal system (Olympus, Japan) after staining with DAPI and FITC‐Phalloidin.

### 2.11 Radiation‐Induced Intestinal Injury Mice Mode and sEV Administration

The radiation‐induced intestinal injury model was established as described (Wei, Yu, Zhang, et al. 2023; Zhou et al. 2013). Briefly, C57BL/6 mice were restrained in a custom‐designed lead shielding jig that exposed only the abdominal area (approximately from the xyphoid process to the pelvis). The head, thorax, limbs, and tail were completely shielded by lead to protect from radiation exposure. This ensures that the observed effects are primarily due to gastrointestinal injury. Irradiation was performed using an RS2000 biological irradiator (Rad Source Technologies) with a dose rate of 2.0 Gy/min. The voltage and current settings were 160 kV and 12.5 mA, respectively. To minimize animal stress and ensure precise positioning during the procedure, all mice were anesthetized using isoflurane before the irradiation. Following irradiation, mice received supportive care including a softened diet, soft bedding, and hydration to minimize suffering and comply with animal welfare guidelines. For rescue experiments, mice were orally administered ∼2 × 10^10^ particles sEV (200 µL, ∼1 × 10^11^ particles/mL) daily for 5 days. Animals were assessed at designated time points after injury.

### 2.12 Ageing Mice and sEV Administration

18‐month‐old C57BL/6J mice were treated with ∼2 × 10^10^ particles sEV (200 µL, ∼1 × 10^11^) particles of sEV by oral administration twice a week for 2 weeks. Mice were euthanized after the last sEV administration for 24 h. The 2‐month‐old C57BL/6J mice were used as young controls.

### 2.13 Real‐Time PCR

Total RNA was extracted from HEK293T cells, intestinal organoids, or part of the mice's intestinal tissues using TRIzol reagent (Invitrogen, Thermo Fisher, USA) and quantified by Nanodrop (Thermo Fisher, USA). Genomic DNA was removed, and cDNAs were synthesized using the HiScript II 1st Strand cDNA Synthesis Kit (+gDNA wiper) (Vazyme, China). Samples were then used for real‐time PCR with SYBR Green Mix (Vazyme, China), and gene expression was normalized to β‐actin. Primers were obtained from Sangon Biotech. (China), and the sequences are listed in Table S1.

### 2.14 RNA‐seq

mRNA sequencing was performed by Genome (China). For short, RNA integrity was assessed using the RNA Nano 6000 Assay Kit of the Bioanalyzer 2100 system (Agilent Technologies). mRNA was purified from total RNA using poly‐T oligo‐attached magnetic beads. After fragmentation, the first strand cDNA was synthesized using random hexamer primers, followed by the second strand cDNA synthesis. The library was checked with Qubit and real‐time PCR for quantification and a bioanalyzer for size distribution detection. Sequencing was performed on an Illumina Novaseq platform, and 150 bp paired‐end reads were generated. Clean data/reads were generated from raw data/reads using fastp software, and mapped to the reference genome using Hisat2 (v2.0.5).

Sequencing data analysis was performed using the DESeq2 package (v1.44.0) in the R environment (v4.4.0). Sample variability was performed by principal component analysis (PCA) using the top 20,000 genes. Differential gene expression analysis was performed using the DESeq2 package and visualized by the ComplexHeatmap package (v4.4.0). Gene set enrichment analysis (GSEA) and pathway enrichment were performed using the cluster Profiler package (v4.12.2) against the KEGG gene sets or gene ontology (GO) gene sets. All data analysis and visualization were performed using R Studio (v2024.04.0).

### 2.15 Western blot

Total protein was extracted from sEV, HEK293T cells, and intestinal organoids using a RIPA lysis buffer (Sangon, China) supplemented with protease inhibitors (Beyotime, China). Protein concentration was quantified using the BCA assay kit and denatured with 5× loading buffer (Beyotime, China) at 100°C for 10 min. About 30 µg of each protein sample was loaded into a 4%–20% SDS‐PAGE gel. After electrophoresis, proteins were transferred onto a PVDF membrane (Millipore, Germany) and blocked with 5% non‐fat milk. Membranes were incubated with primary antibody overnight at 4°C. After washing thrice with TBST solution, membranes were incubated with HRP‐conjugated secondary antibody for 1 h at room temperature. The signal was detected using a High Sensitivity ECL Substrate Kit (Abcam, USA). β‐actin was used as a loading control for the cell lysates, and CD63 or HSP70 was used as a loading control for the sEV lysates. Antibody information was listed in Table S2.

### 2.16 Immunofluorescence

After mouse euthanasia, the intestinal tissues were separated and fixed in 4% PFA. For cryopreservation, intestinal tissues were washed thrice with PBS, immersed in 15% sucrose solution overnight, and transferred to and kept in 30% sucrose solution until they settled to the bottom. The tissues were then embedded in OCT solution and stored at −80°C. Intestinal tissues were sectioned at 5 µm thickness using a cryostat (Leica, Germany) and allowed to defrost and dry at room temperature for 30 min. The sections were dipped in PBS for 10 min and permeabilized using 0.3% Triton‐X100 for 10 min. The sections were rinsed thrice in PBS and blocked with 3% normal sheep serum for 30 min. Then, the sections were incubated with primary antibodies at 4°C overnight. After three washes with PBS, samples were incubated with the corresponding fluorescent‐labelled secondary antibodies for 2 h at room temperature, protected from light. The sections were finally washed with PBS and incubated with DAPI for 5 min. The fluorescence images were captured using confocal microscopy.

### 2.17 H&E Staining

Mice intestinal sections were deparaffinized in xylene and rehydrated through an ethanol gradient. After hydration in distilled water, sections were immersed in hematoxylin for 1 min, rinsed in running tap water, and differentiated in Scott's tap water for 3 min. After being rinsed in tap water, the sections were stained with Eosin‐Y for 3 min and dehydrated in absolute alcohol. The images were scanned by a slide viewer (Olympus, Japan). The dead/non‐surviving crypt was defined as a crypt structure that exhibited any of the following characteristics: fewer than 10 cells, lack of a defined lumen, excessive apoptosis, or severe architectural disruption.

### 2.18 β‐Galactosidase Staining

The ageing marker β‐Galactosidase staining on the mouse intestinal tissues was performed according to the manufacturer's instructions (Servicebio, China). In brief, the frozen sections were rewarmed at room temperature for 10 min and incubated with β‐Galactosidase staining solution at room temperature for 20 min. After three washes with PBS, the sections were incubated with the solution at 37°C for 2 h. Rinsed the sections with PBS and water twice, respectively. The sections were stained with Eosin‐Y for 3 min and dehydrated in absolute alcohol. The images were scanned by a slide viewer (Olympus, Japan).

### 2.19 Image Analysis and Statistical Analysis

ImageJ was used for image analysis as well as quantification. 5–8 view fields in each mouse intestine section were randomly selected and analysed. The data presented are the average counts from these fields. GraphPad Prism 9 was used for statistical analysis. All experiments were performed at least three times, in triplicate, and the statistical data are represented as mean ± SD. Two‐tailed unpaired Student's *t tests* were used to compare the two groups. The *p* values are calculated and evaluated for statistical significance (*ns p* ≥ 0.05; *p < 0.05; **p < 0.01; ***p < 0.001; ****p < 0.0001).

## 3 Results

### 3.1 Active RSPO1 Protein is Loaded on the Surface of sEV via HSPGs

**FIGURE 1:**
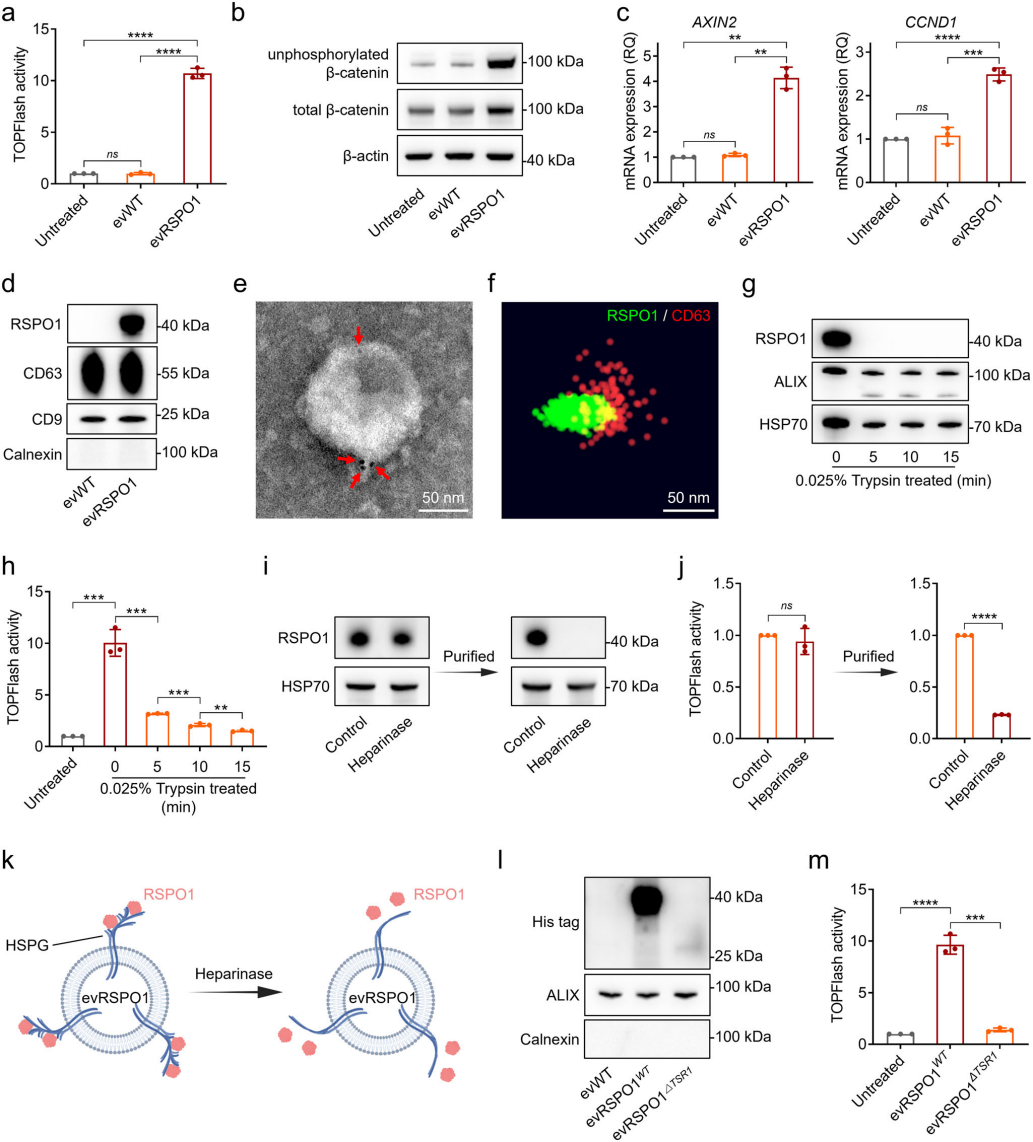
sEV carries active RSPO1 protein on the surface by HSPGs. (a) TOPFlash report activity in HEK293T cells treated with 1 × 10^9^ particles/mL evWT or evRSPO1 for 24 h. The relative activity normalized to the untreated group is shown (*n* = 3). (b) Western blot analysis of unphosphorylated β‐catenin and total β‐catenin protein level in HEK293T cells treated with 1 × 10^9^ particles/mL evWT or evRSPO1 for 24 h. β‐actin was used as a loading control. (c) Real‐time PCR analysis of the expression level of WNT/β‐catenin signalling downstream genes *AXIN2* and *CCND1* in HEK293T cells treated with 1 × 10^9^ particles/mL evWT or evRSPO1 for 24 h. β‐actin was used as a control gene, and the relative fold‐change normalized to the untreated group is shown (*n* = 3). (d) Western blot analysis of RSPO1 protein level on the same particles evWT and evRSPO1. CD9 and CD63 were used as sEV markers and loading controls, Calnexin was used as sEV negative content marker. (e) Immunogold labelling of RSPO1 localization on sEV with anti‐RSPO1 antibody labelled with 10 nm gold nanoparticles (red arrow). (f) dSTROM system detection of RSPO1 protein (green) and CD63 (red) on the sEV with anti‐RSPO1 antibody and anti‐CD63 antibody. (g) Western blot analysis of RSPO1 protein level in evRSPO1 treated with 0.025% Trypsin at room temperature for different periods. ALIX and HSP70 were used as sEV internal protein marker controls. (h) TOPFlash report activity in HEK293T cells treated with evRSPO1 treated with 0.025% Trypsin at room temperature for different periods. The relative activity normalized to the untreated group is shown (*n* = 3). (i) Western blot analysis of RSPO1 protein level in evRSPO1 treated with 10 U/L Heparinase I/III at 37°C for 1.5 h and further purified by EXODUS before used. HSP70 was used as a loading control. (j) TOPFlash report activity of evRSPO1 treated with 10 U/L Heparinase I/III at 37°C for 1.5 h, and further purified by EXODUS before used. The relative activity normalized to the untreated group is shown (*n* = 3). (k) Schematic illustration of Heparinase I/III degraded heparin of HSPGs and released RSPO1 protein from evRSPO1. (l) Western blot analysis of His‐tag expression level in sEV derived from cells expressing full‐length RSPO1 (evRSPO1*^WT^*) or RSPO1*^ΔTSR1^* (evRSPO1*^ΔTSR1^*). ALIX was used as a loading control, and Calnexin was used as an sEV negative protein control. (m) TOPFlash report activity in HEK293T cells treated with 1 × 10^9^ particles/mL evRSPO1*^WT^* and evRSPO1*^ΔTSR1^* for 24 h. The relative activity normalized to the untreated group is shown (*n* = 3). All statistical data are presented as mean ± SD. Two‐tailed unpaired Student's *t* tests are used to compare the two groups. The *p* values are calculated and shown as: *ns p* ≥ 0.05, **p < 0.01, ***p < 0.001, ****p < 0.0001. *n* indicates biological replicates.

We first isolated sEV from conditioned media of RPSO1‐overexpressing HEK293 cells (evRSPO1) and wild‐type HEK293 cells (evWT) (Figure S1a). Both evWT and evRSPO1 exhibited bilayer‐membrane structure with diameters <200 nm (Figure S1b,c). Using the T cell factor (TCF)‐optimized promoter‐luciferase (TOPFlash) reporter assay—a standard method for monitoring canonical WNT/β‐catenin activity (de Lau et al. 2011)—we found that these sEV elicited significantly higher WNT/β‐catenin signalling activity compared to the evWT (Figure 1a). Following evRSPO1 treatment, both unphosphorylated β‐catenin and total β‐catenin were increased in HEK293T cells (Figure 1b), indicating robust β‐catenin stabilization. Correspondingly, canonical WNT/β‐catenin signalling target genes (*AXIN2*, *CCND1*, *TCF4*) and proliferation marker gene (*Ki67*) were substantially upregulated (Figure 1c and Figure S1d). Consistent with this strong WNT‐signalling induction, Western blot analysis confirmed abundant RSPO1 loading on sEV (Figure 1d).

To reveal the localization of the RSPO1 on sEV, we employed immunogold labelling and Stochastic Optical Reconstruction Microscopy (STORM) with a specific anti‐RSPO1 antibody under non‐permeabilizing conditions. Super‐resolution imaging revealed that the RSPO1 is localized on the surface of evRSPO1 (Figure 1e,f). This surface localization was further validated by trypsin digestion, which cleaves membrane surface proteins. RSPO1 was undetected post‐treatment, whereas internal protein markers HSP70 and ALIX remained intact even after extended incubation (Figure 1g). Concomitantly, the TOPFlash activity induced by evRSPO1 was significantly impaired after trypsin treatment (Figure 1h), confirming that surface‐bound RSPO1 is critical for its functional activity.

HSPGs are known to bind cytokines, chemokines, and other growth factors, protecting them from proteolysis (Zhai et al. 2023). To further understand how RSPO1 was attached to the vesicle surface, evRSPO1 was treated with heparinase I/III, which degrades HSPGs into oligosaccharide or unsaturated disaccharide (Li et al. 2020). Notably, heparinase treatment significantly reduced RSPO1 protein levels in evRSPO1 (Figure 1i) and attenuated TOPFlash activity (Figure 1j), indicating that HSPGs degradation releases RSPO1 from the sEV surface (Figure 1k). Furthermore, quantitative liquid chromatography‐tandem mass spectrometry (LC‐MS/MS) revealed 17 distinct HSPGs that could interact with RSPO1 (Figure S1e). The RSPO1 protein domains include two amino‐terminal furin‐like repeats, which are responsible for WNT signal potentiation, and a thrombospondin type 1 (TSR1) domain, which has been reported to have affinity for HSPGs (Chang et al. 2016). To investigate the binding mechanism of RSPO1 to HSPGs on the evRSPO1, we generated cells overexpressing the full‐length RSPO1 (RSPO1*^WT^*‐His) or the TSR1 domain‐depleted RSPO1 variant (RSPO1*^ΔTSR1^*‐His) (Figure S1f,g). EVs were subsequently isolated from these two cells, and protein levels on the sEV were measured. The results demonstrated that the RSPO1*^ΔTSR1^* was scarcely loaded on the sEV, compared to the full‐length RSPO1*^WT^* protein (Figure 1l). Additionally, sEV derived from RSPO1*^ΔTSR1^* cells (evRSPO1*^ΔTSR1^*) exhibited almost no significant TOPFlash activity (Figure 1m). Taken together, these results demonstrated that RSPO1 is actively loaded onto the outer membrane of sEV via HSPGs interaction mediated by its TSR1 domain, and that the surface‐bound evRSPO1 efficiently activates the WNT/β‐catenin signalling.

### 3.2 Scalable Production of evRSPO1 With Superior Protein Stability

**FIGURE 2:**
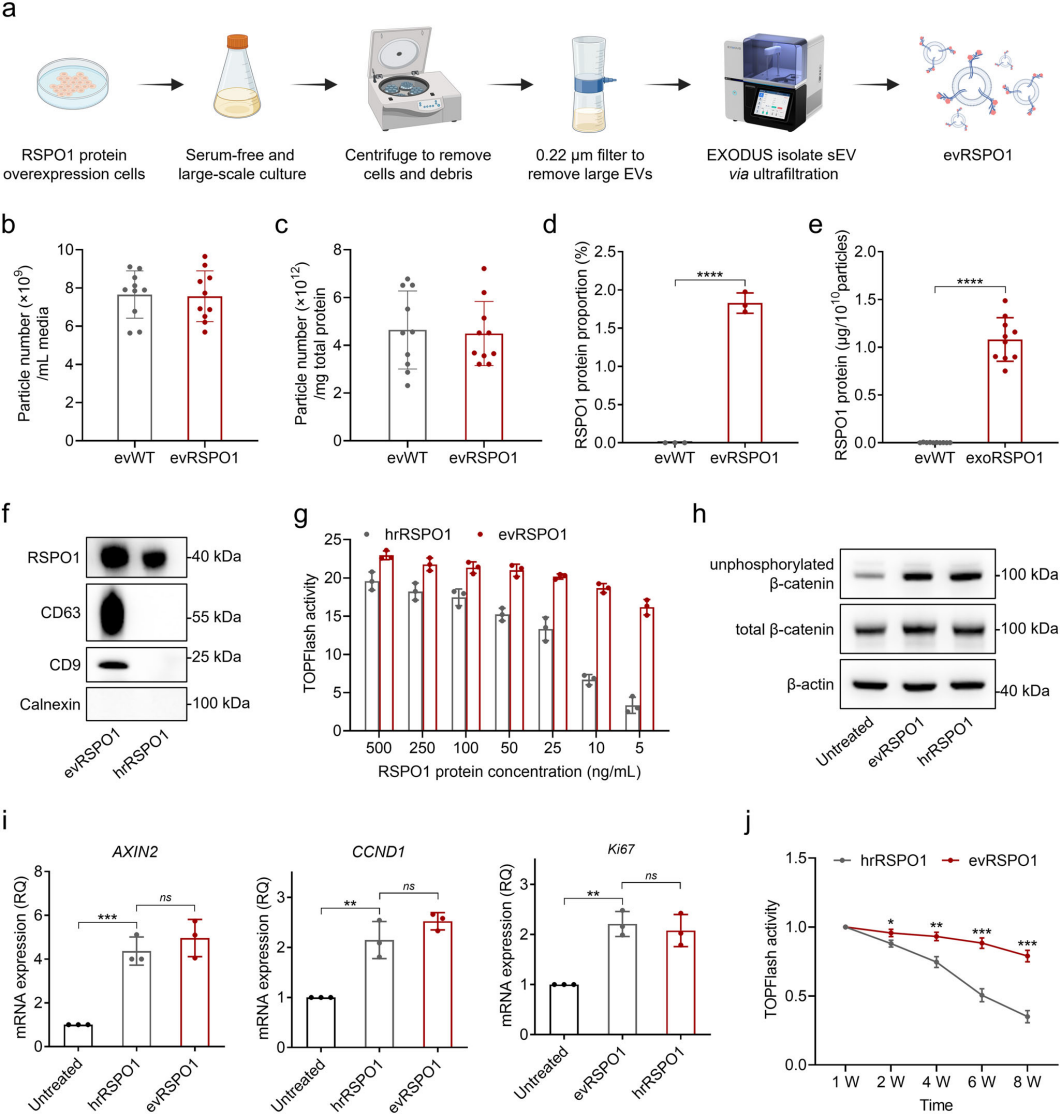
Serum‐free suspension culture and auto‐isolation produce evRSPO1 with high activity and stability. (a) Schematic illustrating the workflow of the evRSPO1 purification from cell culture supernatant. (b) The particle number of sEV was obtained from per mL supernatant (*n* = 10). The particle number was measured using nano‐flow cytometry (Nano‐FCM). (c) Proportion of vesicles in the purified sEV solution. The total protein amount is measured using the BCA assay, and the particle number is detected by Nano‐Flow cytometry (*n* = 10). (d) Statistical analysis of the proportion of RSPO1 protein in evWT and evRSPO1 detected by LC‐MS/MS (*n* = 3). (e) The average level of RSPO1 protein in each 1 × 10^10^ particles sEV (*n* = 10). The RSPO1 protein amount was measured using enzyme‐linked immunosorbent assay (ELISA), and the particle number was measured using nano‐flow cytometry (Nano‐FCM). (f) Western blot analysis of the RSPO1 protein level in 1 × 10^9^ particles/mL evRSPO1 (∼100 ng/mL RSPO1 protein) and 100 ng/mL hrRSPO1. CD9 and CD63 were used as sEV markers and loading controls, and Calnexin was used as sEV negative content marker. (g) TOPFlash reporter assay in HEK293T treated with evRSPO1 and hrRSPO1 under different RSPO1 protein level. The relative activity normalized to the control untreated group is shown (*n* = 3). (h) Western blot analysis of unphosphorylated β‐catenin and total β‐catenin level in HEK293T cells treated with 1 × 10^9^ particles/mL evRSPO1 and 100 ng/mL hrRSPO1 for 24 h. β‐actin was used as a loading control. (i) Real‐time PCR analysis of the expression level of WNT/β‐catenin signalling downstream genes *AXIN2* and *CCND1*, and cell proliferation gene *Ki67* in HEK293T cells treated with 1 × 10^9^ particles/mL evRSPO1 and 100 ng/mL hrRSPO1 for 24 h. β‐actin was used as a control gene, and the relative fold‐change normalized to the untreated group is shown (*n* = 3). (j) TOPFlash reporter assay in HEK293T treated with 1 × 10^9^ particles/mL evRSPO1 and 100 ng/mL hrRSPO1, stored at 4°C for different periods (1 to 8 weeks). The relative activity normalized to the untreated control group is shown (*n* = 3). All statistical data are presented as mean ± SD. Two‐tailed unpaired Student's *t* tests are used to compare the two groups. The *p* values are calculated and shown as: *ns p* ≥ 0.05, *p < 0.05, **p < 0.01, ***p < 0.001, ****p < 0.0001. *n* indicates biological replicates.

To enable large‐scale and high‐purity production for industrial and clinical applications, we established a scalable workflow for culturing RSPO1‐engineered HEK293 cells in a chemically defined, animal‐source‐free medium under suspension conditions. Live cell staining showed that the cell density reached 1 × 10^7^ cells/mL with >95% cell vitality over 72 h of culture. After removal of cellular debris and larger vesicles, sEV were isolated using the ultrafast‐isolation method (EXODUS) (Chen et al. 2021) (Figure 2a). Approximately 7.6 × 10^9^ particles could be obtained per mL supernatant (Figure 2b), with a particle‐to‐protein ratio exceeding 10^12^ particles/mg (Figure 2c), indicating that our workflow could achieve high sEV yield and purity. LC‐MS/MS analysis revealed negligible RSPO1 in evWT (∼0.00028% RSPO1 of total protein), whereas RSPO1 is the most abundant protein in evRSPO1 (∼1.83% RSPO1 of total protein) (Figure 2d and Figure S2a,b). ELISA analysis further showed that 1 × 10^10^ evRSPO1 particles carried approximately 1 µg of RSPO1 on average (Figure 2e), and Western blot confirmed consistent protein loading efficacy relative to hrRSPO1 (Figure 2f).

Functionally, at equivalent protein concentrations, 1 × 10^9^ particles/mL evRSPO1 (∼100 ng/mL RSPO1) and 100 ng/mL hrRSPO1 induced comparable TOPFlash activity (Figure 2g), upregulated both unphosphorylated β‐catenin and total β‐catenin protein levels (Figure 2h), and activated canonical WNT targeting genes, including *AXIN2*, *CCND1*, and proliferation marker gene *Ki67* (Figure 2i). Intriguingly, evRSPO1 treatment elicited more pronounced TOPFlash activity than hrRSPO1 treatment at lower protein titers (Figure 2g). Furthermore, RSPO1 loaded in evRSPO1 retained more than 80% of its activity even after 8 weeks of storage at 4°C, whereas the activity of hrRSPO1 declined significantly over the same period (Figure 2j). These findings demonstrate the feasibility of generating RSPO1‐carrying sEV with high bioactivity and stability—key properties for therapeutic applications.

### 3.3 evRSPO1 Promotes Intestinal Organoids Growth

**FIGURE 3:**
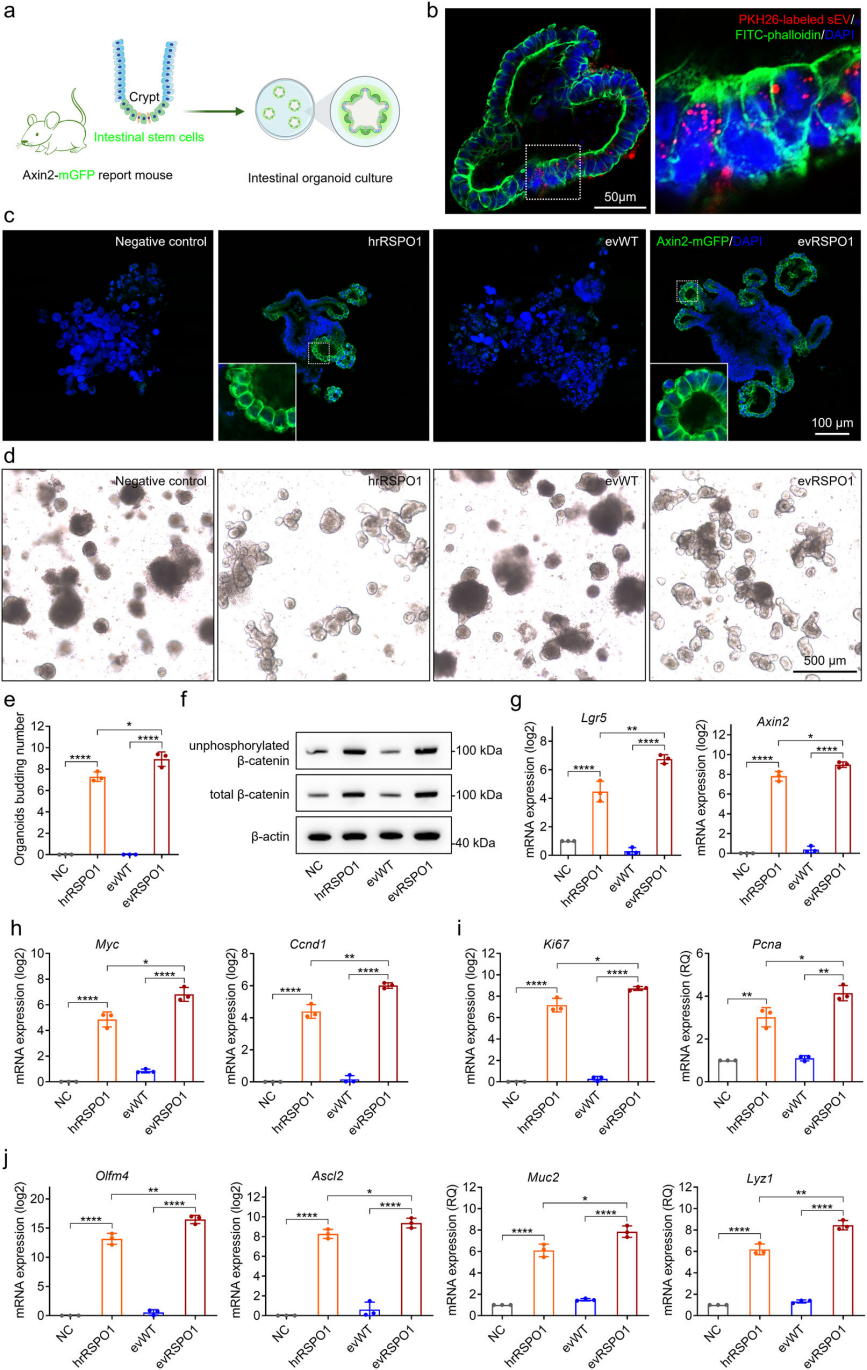
evRSPO1 supported intestinal organoids growth in vitro through activating WNT/β‐catenin signalling. (a) Schematic illustrating the Axin2‐mGFP positive intestinal organoids derived from Axin2‐mGFP mice. (b) Fluorescent images showing the cellular uptake of PKH26‐labelled sEV by intestinal organoids within 12 h. (c) Fluorescent images showing the Axin2‐mGFP signals in crypt‐villus structures of intestinal organoids treated with negative control without hrRPSO1, 100 ng/mL hrRSPO1, 1 × 10^9^ particles/mL evWT, or evRSPO1 for 48 h. (d) Representative images showing the crypt‐villus structures in intestinal organoids treated with negative control, 100 ng/mL hrRSPO1, 1 × 10^9^ particles/mL evWT, or evRSPO1 for 48 h. (e) Quantification of the number of crypt‐villus structures in intestinal organoids from (d). (f) Western blot analysis of unphosphorylated β‐catenin and total β‐catenin protein level in intestinal organoids treated with negative control, 100 ng/mL hrRSPO1, 1 × 10^9^ particles/mL evWT, or evRSPO1 for 48 h. (g) Real‐time PCR analysis of the expression level of WNT/β‐catenin signalling downstream genes *Lgr5* and *Axin2* in intestinal organoids treated with 100 ng/mL hrRSPO1, 1 × 10^9^ particles/mL evWT, or evRSPO1 for 48 h. β‐actin was used as a control gene, and the relative fold‐change normalized to the untreated group is shown (*n* = 3). (h) Real‐time PCR analysis of the expression level of WNT/β‐catenin signalling downstream genes *Myc* and *Ccnd1* in intestinal organoids treated with 100 ng/mL hrRSPO1, 1 × 10^9^ particles/mL evWT, or evRSPO1 for 48 h. β‐actin was used as a control gene, and the relative fold‐change normalized to the untreated group is shown (*n* = 3). (i) Real‐time PCR analysis of the expression level of cell proliferation marker gene *Ki67* and *Pcna* in intestinal organoids treated with 100 ng/mL hrRSPO1, 1 × 10^9^ particles/mL evWT, or evRSPO1 for 48 h. β‐actin was used as a control gene, and the relative fold‐change normalized to the untreated group is shown (*n* = 3). (j) Real‐time PCR analysis of the expression level of *Olmf4*, *Ascl2, Muc2*, and *Lyz1* in intestinal organoids treated with 100 ng/mL hrRSPO1, 1 × 10^9^ particles/mL evWT, or evRSPO1 for 48 h. β‐actin was used as a control gene, and the relative fold‐change normalized to the untreated group is shown (*n* = 3). All statistical data are presented as mean ± SD. Two‐tailed unpaired Student's *t* tests are used to compare the two groups. The *p* values are calculated and shown as: *p < 0.05, **p < 0.01, ****p < 0.0001. *n* indicates biological replicates.

Intestinal organoids culture requires activation of WNT/β‐catenin signalling to form crypt‐villus structures (Sato et al. 2009), making intestinal organoids an ideal ex vivo model to evaluate evRSPO1 capacity to promote intestinal regeneration. *Axin2*, a well‐characterized ISC marker, is transcriptionally activated by WNT signalling (Wang et al. 2021). To assess the effect of evRSPO1, organoids were generated from the intestinal crypts harvested from Axin2‐mGFP mice (Figure 3a). Using PKH26‐labelled sEV, we observed efficient uptake of evRSPO1 by intestinal organoids within 12 h (Figure 3b). As shown in Figure 3c, both hrRSPO1 and evRSPO1 markedly induced strong Axin2‐mGFP signals in intestinal organoids, compared to the negative control (no RSPO1) and treated with evWT (Figure 3c). Notably, evRSPO1 treatment significantly enhanced intestinal organoids bud formation (Figure 3d,e), and upregulated the protein expression of both total β‑catenin and unphosphorylated β‑catenin (Figure 3f). Consequently, expression of WNT/β‑catenin signalling target genes, including *Lgr5*, *Axin2*, *Myc*, and *Ccnd1*, was also elevated (Figure 3g,h). Of note, cell proliferation‐associated genes (*Ki67* and *Pcna*) were more highly expressed in evRSPO1‐treated organoids than in hrRSPO1‐treated groups (Figure 3i), highlighting evRSPO1 as a superior alternative to hrRSPO1.

To assess the cell type composition of intestinal organoids under different treatments, real‐time PCR analysis was performed to detect marker genes, including ISCs markers *Olfactomedin‐4* (*Olfm4*), *Achaete Scute‐Like 2* (*Ascl2*), intestinal goblet cells marker *Mucin 2* (*Muc2*), and mature Paneth cells marker *lysozyme1* (*Lyz1*) (Figure 3j). The results revealed that evRSPO1‐treated intestinal organoids maintained the major epithelial cell types.

**FIGURE 4:**
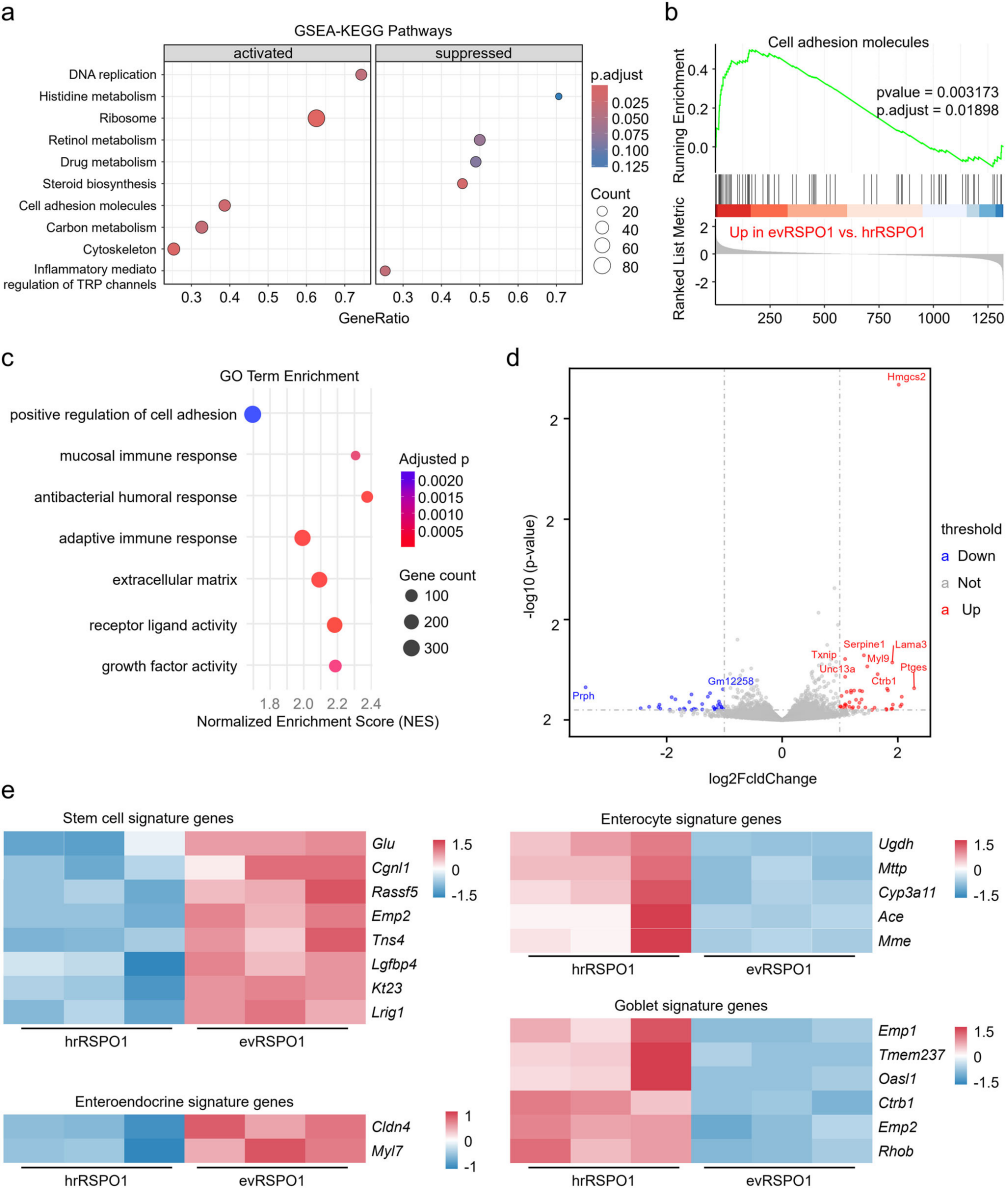
evRSPO1 enhanced DNA replication, cell adhesion, and stem cell gene expression in intestinal organoids. (a) GSEA‐KEGG pathway enrichment analysis of transcriptomic profiles comparing evRSPO1‐ versus hrRSPO1‐treated intestinal organoids. Gene ranking was performed based on log2 fold change derived from RNA‐seq data. Significantly enriched pathways (FDR < 0.05) are categorized by regulation direction (NES > 0: activated in evRSPO1‐treated group; NES < 0: suppressed in evRSPO1‐treated group) with point size proportional to gene set size and color intensity denoting statistical significance (‐log10[adjusted *p* value]). The top 5 enriched pathways per category are shown. (b) GSEA reveals significant enrichment of the cell adhesion molecules pathway in evRSPO1‐treated intestinal organoids. (c) Gene ontology (GO) enrichment analysis of transcriptomic profiles comparing evRSPO1‐ versus hrRSPO1‐treated intestinal organoids, visualized through a faceted dot plot. Points represent individual GO terms, with color intensity indicating the statistical significance of enrichment (‐log10[adjusted *p* value]; red: highest significance, blue: lowest) and point size scaled to the number of enriched genes. The Normalized Enrichment Score (NES) quantifies pathway activation direction (NES > 0: upregulated in evRSPO1; NES < 0: suppressed). (d) Volcano plot displaying differentially expressed genes (DEGs) between evRSPO1‐ and hrRSPO1‐treated intestinal organoids. (e) Heatmap depicting expression patterns of stem cell and differentiated cell signature genes across experimental conditions.

Transcriptome of evRSPO1‐ and hrRSPO1‐treated intestinal organoids was profiled to reveal further phenotypic differences. PCA showed that evRSPO1‐ and hrRSPO1‐treated groups exhibited convergent gene expression signatures compared to the negative control and evWT‐treated group (Figure S4a). GSEA revealed that evRSPO1‐upregulated genes were enriched in pathways related to cell adhesion molecules and cytoskeletal reorganization (Figure 4a,b). GO analysis further indicated that evRSPO1 enhanced the expression of genes involved in receptor‐ligand activity and growth factor binding, implicating its capacity to remodel niche signalling (Figure 4c). The volcano plot further highlighted key evRSPO1‐upregulated effectors: *Hmgcs2* (which drives ketogenesis‐mediated metabolic reprogramming to sustain stemness through β‐hydroxybutyrate‐dependent epigenetic remodelling) (Cheng et al. 2019) and *Ptges* (which promotes crypt regeneration through prostaglandin E2/EP2‐EP4 axis activation) (Wei, Li, Song, et al. 2023) (Figure 4d). Consistently, comparative analysis revealed that evRSPO1 treatment upregulated stem cell and enteroendocrine signatures while suppressing enterocyte and goblet cell markers (Figure 4e). Of note, stem cell‐associated genes enriched in the hrRSPO1‐treated group were further elevated in the evRSPO1‐treated group (Figure S4b). This included markers of several ISC populations (including revival stem cell, active ISC, and quiescent ISC) (Figure S3c–e), suggesting that evRSPO1 strongly induced WNT‐dependent stemness programs in the organoids. Overall, these results highlight the potency of evRSPO1 to modulate ISC proliferation and crypt formation, supporting its potential for intestinal regeneration.

### 3.4 Orally Delivered evRSPO1 Activates WNT/β‐Catenin Signalling in Mouse Intestinal Tissue

**FIGURE 5:**
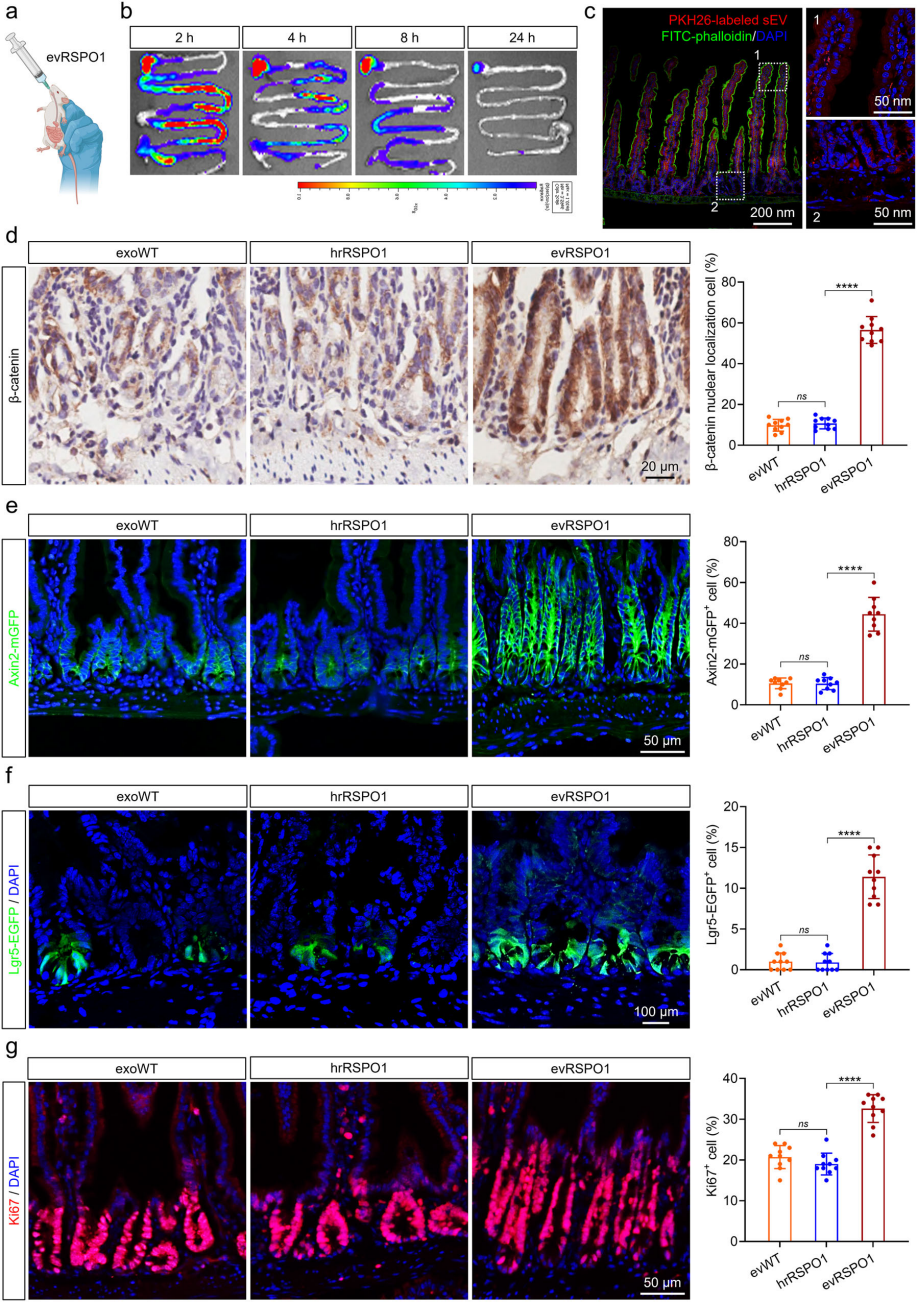
Oral administration of evRSPO1 activated the WNT/β‐catenin signalling pathway in mouse intestinal and promoted ISCs proliferation. (a) Schematic diagram of delivery of evRSPO1 to the mouse intestine through oral administration. (b) Fluorescence images showing the biodistribution of DiR‐labelled sEV after oral administration at 2, 4, 8, and 24 h. (c) Fluorescent images of intestinal tissue sections showing the distribution of PKH26‐labelled sEV after oral administration for 8 h. (d) Immunohistochemistry staining showing the nuclear translocation of β‐catenin in intestinal crypt cells after 2 × 10^10^ particles evWT or evRSPO1, or hrRSPO1 oral administration for 24 h. Quantification of the percentage of β‐catenin nuclear translocation positive cells per crypt (*n* = 10). (e) Fluorescent images of intestinal tissue sections showing the Axin2‐mGFP signals in crypt cells after 2 × 10^10^ particles evWT or evRSPO1, or hrRSPO1 oral administration for 24 h. Quantification of the percentage of Axin2‐mGFP positive cells per crypt (*n* = 10). (f) Fluorescent images of intestinal tissue sections showing the Lgr5‐EGFP signals in crypt cells after 2 × 10^10^ particles evWT or evRSPO1, or hrRSPO1 oral administration for 24 h. Quantification of the percentage of Lgr5‐EGFP positive cells per crypt (*n* = 10). (g) Fluorescent images of intestinal tissue sections showing the Ki67 signals in crypt cells after 2 × 10^10^ particles evWT or evRSPO1, or hrRSPO1 oral administration for 24 h. Quantification of the percentage of Ki67‐positive cells per crypt (*n* = 10). All statistical data are presented as mean ± SD. Two‐tailed unpaired Student's *t* tests are used to compare the two groups. The *p* values are calculated and shown as: *ns p* ≥ 0.05, ****p < 0.0001. *n* indicates biological replicates.

To evaluate the capacity of evRSPO1 to induce the activity of WNT/β‐catenin signalling in mouse intestinal tissue, we first assessed evRSPO1 in vivo distribution after oral administration (Figure 5a). 2 × 10^10^ particles DiR‐labelled evWT or evRSPO1 (∼2 µg RSPO1 protein) administered reached the intestine within 2–4 h, with a gradual decline from 4 to 8 h, and was largely cleared after 24 h (Figure 5b). To further assess sEV cellular uptake by intestinal cells, PKH26‐labelled sEV was orally administered, and the PKH26 signals were detected both in the intestinal crypts and villi after 8 h (Figure 5c). Importantly, oral evRSPO1 treatment could robustly induce WNT/β‐catenin signalling in crypt‐resident cells, as evidenced by marked increases in β‐catenin nuclear localization (Figure 5d) and target genes—*Axin2* and *Lgr5*, using Axin2‐mGFP and Lgr5‐EGFP reporter mouse (Figure 5e,f). Importantly, levels of Ki67^+^ crypt cells in the intestinal epithelium significantly increased after evRSPO1 treatment compared to controls (Figure 5g). On the other hand, oral delivery of an equivalent amount of hrRSPO1 protein failed to elicit similar responses (Figure 5d–g), likely due to degradation in the gastric environment. These results confirmed that evRSPO1 enabled oral administration and delivery of functional RSPO1 to intestinal tissue.

To further assess the safety of oral administration of evRSPO1, multiple organs were collected after mice underwent a longer biweekly treatment of up to 4 weeks (2 doses per week). No significant histopathological changes were observed in the heart, liver, spleen, lung, kidney, or intestine after 2 or 4 weeks of treatment, indicating the relative safety of oral evRSPO1 administration within these treatment periods (Figure S4).

### 3.5 evRSPO1 Promotes Tissue Regeneration in Radiation‐Induced Intestinal Injury

**FIGURE 6:**
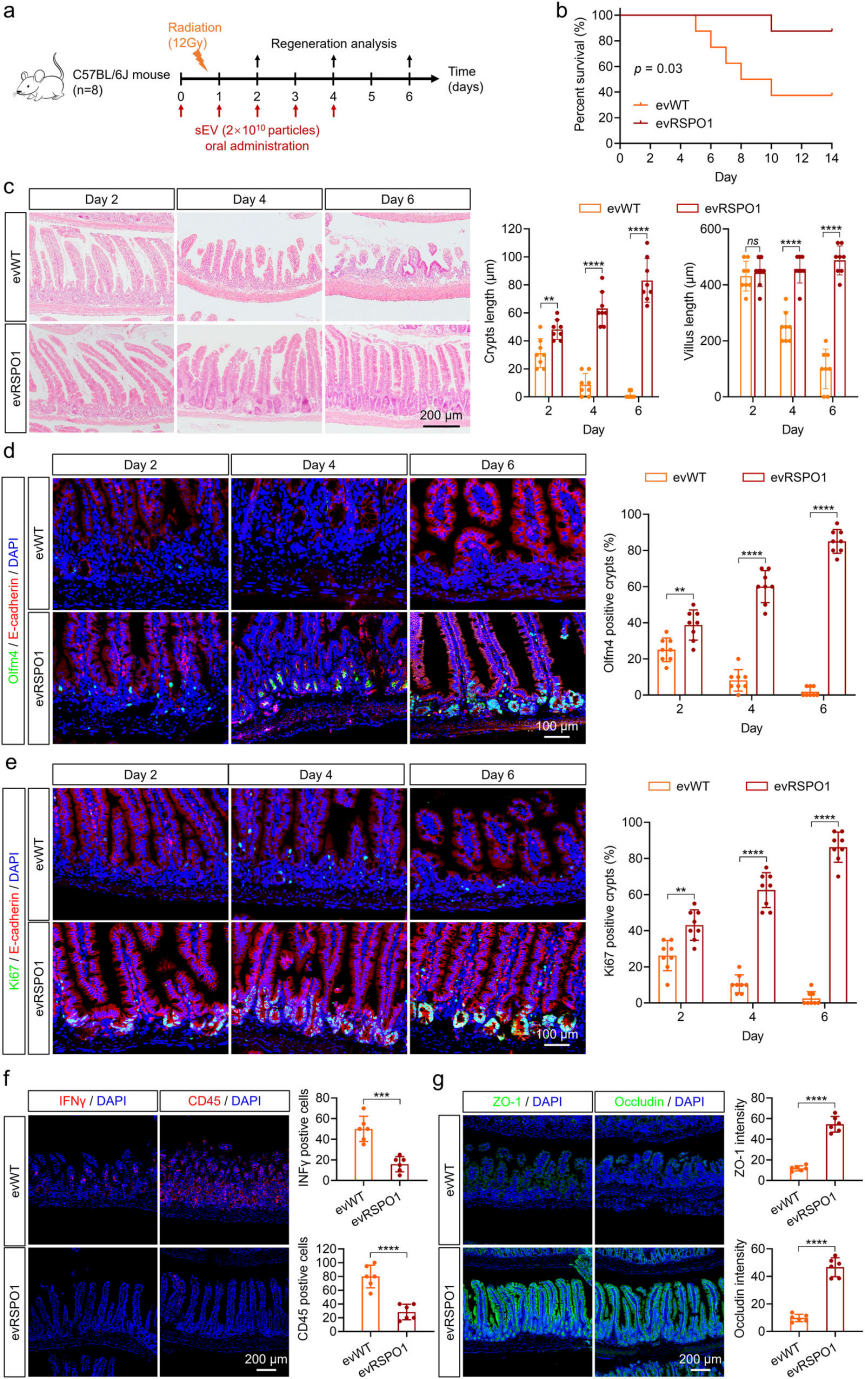
Oral administration of evRSPO1 accelerated intestinal tissue regeneration for radiation‐induced injury. (a) Schematic diagram of the radiation‐induced intestinal injury mouse model and sEV treatment. (b) Percent survival curve of radiation‐induced intestinal injury in mice under 2 × 10^10^ particles evWT or evRSPO1 treated. (c) H&E staining of intestinal tissue from radiation‐induced injury mice under 2 × 10^10^ particles evWT or evRSPO1 treated at Day 2, 4, or 6. Quantification of the length of crypt and villus (*n* = 8). (d) Immunofluorescent staining of Olfm4 (green) and E‐cadherin (red) positive cells in intestinal tissue from radiation‐induced injury mice under 2 × 10^10^ particles evWT or evRSPO1 treated at Day 2, 4, or 6. Quantification of the Olmf4‐positive cells per crypt (*n* = 8). (e) Immunofluorescent staining of Ki67 (green) and E‐cadherin (red) positive cells in intestinal tissue from radiation‐induced injury mice under 2 × 10^10^ particles evWT or evRSPO1 treated at Day 2, 4, or 6. Quantification of the Ki67‐positive cells per crypt (*n* = 8). (f) Immunofluorescent staining of IFNγ (red) and CD45 (red) positive cells in intestinal tissue from radiation‐induced injury mice under 2 × 10^10^ particles evWT or evRSPO1 treated at Day 6. Quantification of the IFNγ and CD45 positive cells (*n* = 6). (g) Immunofluorescent staining of ZO‐1 (green) and Occludin (green) intensity in intestinal tissue from radiation‐induced injury mice under 2 × 10^10^ particles evWT or evRSPO1 treated at Day 6. Quantification of the intensity of ZO‐1 and Occludin (*n* = 6). All statistical data are presented as mean ± SD. Two‐tailed unpaired Student's *t* tests are used to compare the two groups. The *p* values are calculated and shown as: *ns p* ≥ 0.05, **p < 0.001, ****p < 0.0001. *n* indicates biological replicates.

To assess the treatment effect of evRSPO1 in radiation‐induced intestinal injuries, mice received daily oral doses of evWT or evRSPO1 starting 1 day before radiation exposure (12 Gy single‐dose) and continuing for 5 days (Figure 6a). Survival in the evWT‐treated group was 37.5% (3 out of 8 mice) at Day 14, whereas evRSPO1 significantly improved survival to 87.5% (7 out of 8 mice) (Figure 6b). Histological analysis revealed shrunken crypts in evWT‐treated mice 2–4 days post‐irradiation, whereas evRSPO1 accelerated crypt recovery and epithelial regeneration, as evidenced by the increased lengths of both crypts and villi (Figure 6c). In line with enhanced tissue restoration, evRSPO1 prevented the loss of Olm4^+^ ISCs (Figure 6d) and increased Ki67^+^ proliferating cells (Figure 6e). In addition, evRSPO1 reduced inflammatory infiltration, as indicated by decreased CD45^+^ immune cells and IFNγ^+^ cells (Figure 6f). Importantly, strengthened tight junctions, indicated by ZO‐1 and occludin expression, in the evRSPO1‐treated mice suggested that intestinal barrier integrity was rapidly restored after injury (Figure 6g).

### 3.6 Orally Administered evRSPO1 Reverses Senescence Phenotypes in Aged Mice

**FIGURE 7:**
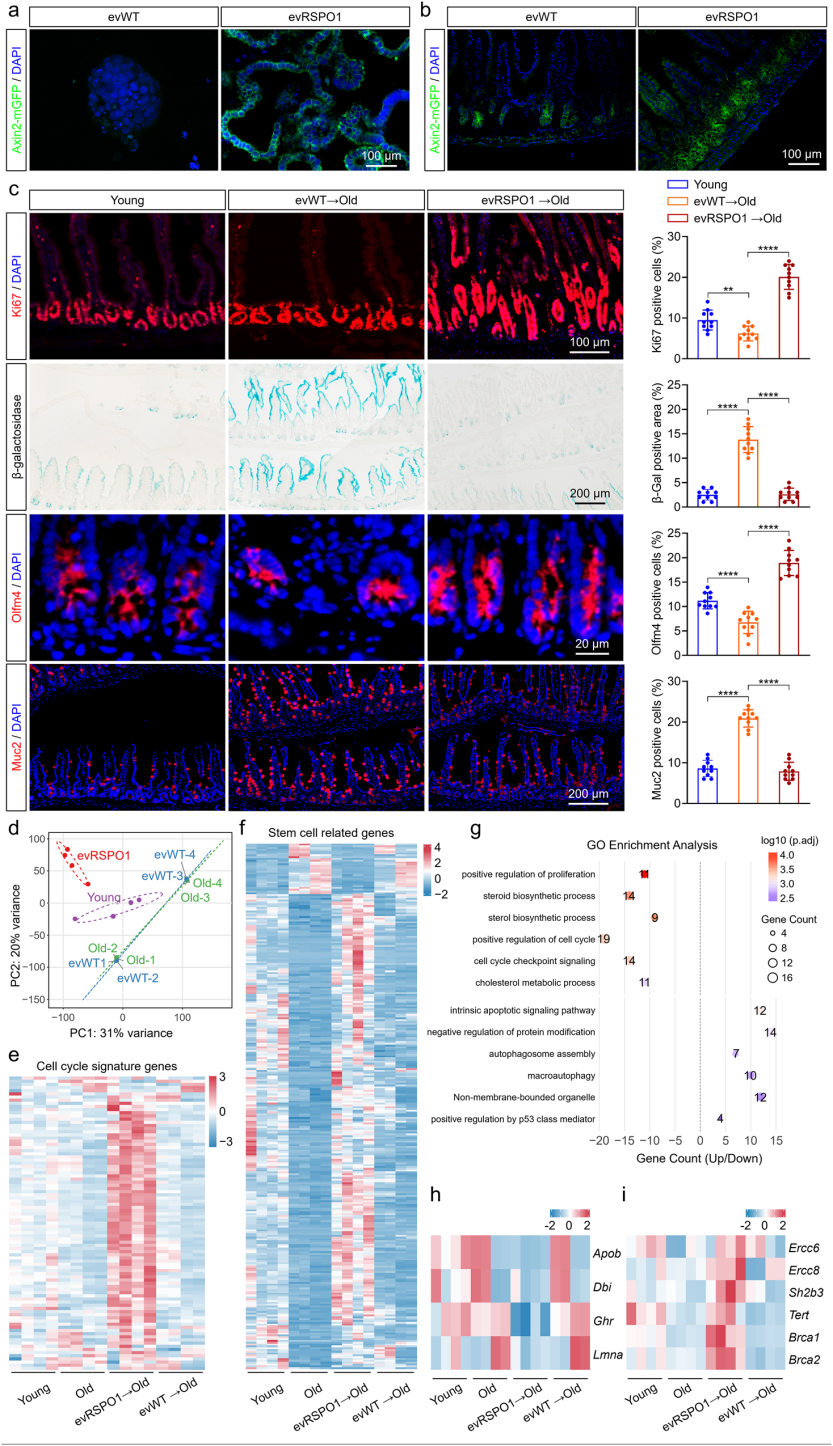
Oral administration of evRSPO1 active WNT signalling pathway and reverse senescence phenotype in ageing mouse intestine. (a) Fluorescent images showing the Axin2‐mGFP signals in old mice‐derived intestinal organoids treated with 1 × 10^9^ particles/mL evWT or evRSPO1 for 48 h. (b) Fluorescent images showing the Axin2‐mGFP signals in old mice intestine after 2 ×10^10^ particles evWT or evRSPO1 oral administration for 24 h. (c) Immunofluorescent staining of Ki67, Olfm4, and Muc2 positive cells and β‐galactosidase staining in the intestinal tissue from young mice and old mice after 2 × 10^10^ particles evWT or evRSPO1 oral administration for 24 h. Quantification of the percentage of the Ki67, Olfm4, and Muc2, and the positive area of β‐galactosidase (*n* = 6). (d) Principal component analysis (PCA) of gene expression profiles, showing sample separation by group. Labels represent individual samples, with ellipses indicating 68% confidence intervals for each group. (e) Heatmap showing cell cycle‐related gene expression patterns in the intestinal tissue from young mice and old mice after 2 × 10^10^ particles evWT or evRSPO1 oral administration for 2 weeks. (f) Heatmap showing stem cell‐related gene expression in the intestinal tissue from young mice and old mice after 2 × 10^10^ particles evWT or evRSPO1 oral administration for 2 weeks. (g) GO enrichment analysis of genes in the intestinal tissue from young mice and old mice after 2 × 10^10^ particles evWT or evRSPO1 oral administration for 2 weeks. The bubble plot shows significantly enriched biological processes (p.adj < 0.01). (h) Heatmap showing four gerogenes expression in the intestinal tissue from young mice and old mice after 2 × 10^10^ particles evWT or evRSPO1 oral administration for 2 weeks. (i) Heatmap showing six Gerosuppressors expression in the intestinal tissue from young mice and old mice after 2 × 10^10^ particles evWT or evRSPO1 oral administration for 2 weeks. All statistical data are presented as mean ± SD. Two‐tailed unpaired Student's *t* tests are used to compare the two groups. The *p* values are calculated and shown as: *ns p* ≥ 0.05, ****p < 0.0001. *n* indicates biological replicates.

Ageing‐associated decline in canonical WNT/β‑catenin signalling reduces intestinal regenerative capacity, and reactivating WNT/β‐catenin signalling in aged ISCs restores function in organoids and improves regeneration of aged ISCs in vitro (Li et al. 2025; Nalapareddy et al. 2017). To investigate the potential of evRSPO1 in counteracting this decline, intestinal organoids were derived from 18‐month‐old mice, which exhibited high expression of the ageing markers *p16* and *p21* (Figure S5a). After evRSPO1 treatment, organoids from aged mice showed robust WNT/β‐catenin signalling reactivation, as evidenced by elevated Axin2‐mGFP expression (Figure 7a and Figure S5b). In vivo, oral administration of evRSPO1 similarly enhanced WNT signalling in aged intestinal crypts, as indicated by increased Axin2‐mGFP signals (Figure 7b and Figure S5c). Strikingly, evRSPO1 increased the cell proliferation‐associated marker Ki67 and reduced the expression of the senescent marker β‐galactosidase, p16, and p21, comparable to those in young mice (Figure 7c and Figure S5d,e). Moreover, evRSPO1 replenished Olfm4^+^ ISCs and normalized the abundance of Muc2^+^ goblet cells, which are characteristic of aged epithelia, to levels seen in young mice (Figure 7c). These results suggested that evRSPO1 not only rejuvenates the stem cells but also rebalances epithelial differentiation.

To determine further the range of cell types affected by orally administered evRSPO1 in aged mouse tissues, a panel of immune cell subsets and fibroblast populations was analysed. Immune and stromal profiling revealed that oral evRSPO1 increased Ly6C^+^ monocytes and TIM‐3 expression, while reducing CD3ε^+^ T cells, CD11B^+^ myeloid cells, and α‐SMA^+^ fibroblasts. CD45R^+^ B cells were preserved, and Collagen I^+^ stromal cells increased compared with evWT controls, suggesting that evRSPO1 indirectly remodels immune and stromal compartments via epithelial restoration (Figure S6).

Transcriptomic PCA revealed clear separation between young and aged mice, with evRSPO1‐treated aged mice clustering closer to young controls (Figure 7d). evRSPO1 reactivated cell cycle‐related genes downregulated during ageing (Figure 7e), and restored expression patterns of stem cell regulatory genes toward youthful levels (Figure 7f). GO analysis highlighted enrichment in proliferative pathways (e.g., fibroblast growth) and metabolic processes (sterol biosynthesis), alongside downregulation of apoptosis, consistent with renewed tissue homeostasis (Figure 7g). Notably, evRSPO1 downregulated pro‐ageing gerogenes, including *Apob* (linked to lipid metabolism dysfunction), *Dbi* (an autophagy suppressor), *Ghr* (growth hormone receptor linked to reduced longevity), and *Lmna* (a nuclear lamina destabilizer) (Figure 7h). Conversely, evRSPO1 activated critical gerosuppressors genes, such as the DNA repair guardians *Brca1*, *Brca2*, *Ercc6*, *Ercc8*, the telomerase component *Tert*, and the longevity‐associated factor *Sh2b3* (Figure 7i). These results indicated the potential of evRSPO1 as a novel gerotherapeutic agent targeting aged intestines.

## 4 Discussion

RSPO1 plays a pivotal role in potentiating canonical WNT/β‐catenin signalling, a pathway critical for maintaining ISC homeostasis and driving tissue regeneration. This makes RSPO1 a promising candidate for regenerative medicine, particularly in treating gastrointestinal disorders characterized by impaired epithelial repair. However, its clinical translation has been hindered by challenges in protein stability, delivery efficiency, and aggregation propensity. In this study, we demonstrated that sEV is a promising vector for delivering functional RSPO1 to the intestine via oral administration. Our findings revealed that RSPO1 interacts with the transmembrane protein HSPGs and is loaded on the outside of evRSPO1. RSPO1 carried on the sEV maintained the stability and efficacy to induce WNT/β‐catenin signalling, compared with the free‐form hrRSPO1. evRSPO1 accelerated ISC proliferation to drive tissue regeneration after radiation‐induced intestinal injury and reversed age‐associated intestinal senescence.

sEV have been reported to reach the intestine after oral administration and exert therapeutic effects. For instance, milk‐derived sEVs contain large amounts of immunomodulatory proteins that play crucial roles in regulating intestinal immunity, remodelling the gut microbiota, and safeguarding intestinal barrier integrity (Tong et al. 2023). In parallel, Roseburia intestinalis‐derived sEVs have been shown to strengthen the intestinal barrier, alleviate colonic inflammation, and facilitate tissue repair in dextran sulfate sodium (DSS)‐induced colitis models (Han et al. 2024). Advancements in sEV engineering have further expanded their therapeutic potential in gastrointestinal protection. Genetically modified sEVs expressing interleukin 10 have been shown to alleviate inflammation and promote colonic epithelial repair in inflammatory bowel disease (Liu et al. 2023). Additionally, bioengineered sEV‐like nanoparticles derived from ginger, when prebound to probiotics, enhance probiotic uptake efficiency, potentiate antibacterial activity, and maintain gut microbiota homeostasis (Pan et al. 2025). These findings highlight the versatility of sEVs as promising therapeutic agents for gastrointestinal disorders. evRSPO1 exemplifies a targeted and potent therapeutic strategy by combining RSPO1‐driven WNT activation with sEV‐mediated delivery to promote intestinal regeneration in disease or injured conditions.

Ageing impairs ISCs’ regenerative capacity, correlating with systemic metabolic disorders and immune senescence (Zhang et al. 2021). Our findings demonstrated that evRSPO1‐mediated LGR5 agonism restored canonical WNT signalling in senescent crypts, thereby rescuing the regenerative capacity of ISCs, aligning with prior evidence that WNT attenuation drives age‐associated decline in ISCs (Wu et al. 2021). Notably, the transcriptional reprogramming induced by evRSPO1 extends beyond stem cell activation, encompassing stromal and metabolic rejuvenation. For example, the upregulation of *Pdgfra* in evRSPO1‐treated mice reflects the reestablishment of mesenchymal‐epithelial crosstalk, a critical mechanism disrupted during the ageing process (Maimets et al. 2022). By downregulating pro‐ageing gerogenes and activating gerosuppressors, evRSPO1 rebalanced antagonistic pleiotropy—a core ageing driver in which early‐life fitness genes become deleterious in late life. This modulation may mitigate inflammaging and genomic instability while restoring sterol biosynthesis, essential for membrane integrity and signalling fidelity (Franceschi et al. 2018; Lombard et al. 2005).

We observed that RSPO1 was loaded on the outside of sEV via binding to HSPGs, aligning with prior research showing that sEV binds chemokines and cytokines, including CCL2 (Lima et al. 2021) and VEGF (Li et al. 2020; Zhou et al. 2024). HSPGs, which are glycoproteins anchored to the extracellular matrix and cell membranes, play a pivotal role in regulating the bioactivity of soluble protein ligands. They are essential for the spatial control of extracellular signals that govern cellular processes, including growth, survival, and differentiation. Structurally, HSPGs possess a protein core covalently linked to heparan sulfate (HS) polysaccharide chains, with highly modified domains that provide docking sites for diverse bioactive molecules, facilitating ligand immobilization and signalling modulation. Syndecan‐1, a key HSPG in certain cell types, has been shown to regulate WNT signalling (Alexander et al. 2000). Specifically, HS chains on syndecan‐1 enhance autocrine and paracrine WNT signalling (Ren et al. 2018). RSPO1 features two amino‐terminal furin‐like repeats, which are responsible for potentiating WNT/β‐catenin signalling, and a thrombospondin type 1 (TSR1) domain can provide affinity for heparin or HSPG (Ohkawara et al. 2011). In addition, positively charged surface amino acids in the TSR1 domain and C‐terminal regions may contribute to the heparin binding. Significantly, HSPG also has the potential to absorb hydrophobic WNT ligands by HS chains, modulating the amplitude of WNT signalling (Ren et al. 2018). Importantly, surface‐displayed proteins on sEV can directly interact with their receptors on recipient cell membranes, without requiring internal cargo delivery.

In this study, HEK293 cells were selected for the production of evRSPO1 due to their well‐established use in recombinant RSPO1 production and their suitability for clinical‐grade sEV production. A recent study demonstrated the batch stability and in vivo safety of sEV derived from HEK293 cells, highlighting their potential as a safe and reliable drug delivery platform for clinical applications (Chen et al. 2025). Notably, HEK293F cells also excel in high‐density suspension culture and grow in chemically defined, serum‐free media, thereby avoiding contamination from animal‐derived proteins and cell‐free nucleic acids, meeting regulatory authorities' acceptable limits (Vo et al. 2024). These attributes make them particularly well‐suited for large‐scale sEV production at GMP‐grade and quality for drug delivery applications in the clinic.

While this study primarily demonstrates the efficacy of evRSPO1 as a prophylactic agent, our preliminary data showing improved survival with post‐exposure administration highlight its strong potential to be developed as a mitigator for gastrointestinal acute radiation syndrome (GI‐ARS), aligning with the NIAID guidelines. A comprehensive future study to optimize the therapeutic window for evRSPO1 as a mitigator would further validate evRSPO1 as a therapeutic option. In addition, it is important to note that in the current study, we utilized a single‐dose irradiation model to establish proof‐of‐concept for the efficacy of oral evRSPO1 against acute GI‐ARS. Future studies employing clinically relevant fractionated radiation regimens will be essential to fully evaluate its potential as a medical countermeasure in radiotherapy settings.

In summary, our study demonstrates that evRSPO1, produced via a scalable suspension culture and ultrafast‐isolation system process with high yield and purity, exhibits significant therapeutic potential by activating the WNT/β‐catenin signalling pathway. Through oral administration, evRSPO1 not only promotes ISC proliferation to accelerate the injured intestinal tissue regeneration but also rescues the aged intestinal phenotypes. These results establish a solid foundation for advancing evRSPO1 towards clinical translation and therapeutic development.

## Author Contributions

**Lingyan Yang**: writing – original draft, investigation, conceptualization, supervision, project administration, visualization. **Xu Wang**: writing – original draft, methodology, software, conceptualization. **Xiyang Wei**: data curation, software, methodology, conceptualization. **Pei Yu**: formal analysis, data curation. **Yue Liu**: methodology, data curation. **Shixiang Wang**: software, methodology, formal analysis. **Yuefang Lin**: methodology. **Yue Yang**: methodology, formal analysis. **Ting Jiang**: methodology, data curation. **Zhiping Qiao**: methodology, data curation. **Jiaxiang Zhang**: methodology, data curation. **Shicheng Yu**: formal analysis, software. **Ye‐Guang Chen**: writing – review and editing, supervision. **Yun‐Shen Chan**: writing – review and editing, funding acquisition, validation, resources, supervision.

## Conflicts of Interest

The authors declare no conflicts of interest.

## Supporting information




**Supplementary materials**: jev270226‐sup‐0001‐SuppMat.docx.

## Data Availability

The data that support the findings of this study are available from the corresponding author upon reasonable request.
