# Supplementary material for: Oral Delivery of R‐spondin1‐Loaded Small Extracellular Vesicles Activates WNT Signalling Pathway to Accelerate Intestinal Injury Repair and Reverse Ageing
Source: J Extracell Vesicles. 2026 Jan 22;15(1):e70226. doi: 10.1002/jev2.70226 (PMC12825024; doi:10.1002/jev2.70226)
Supplement: Supplementary file 1 — Supplementary materials: jev270226‐sup‐0001‐SuppMat.docx. [file JEV2-15-e70226-s001.docx]

# Supplementary Information

**Oral Delivery of R-spondin1-Loaded Small Extracellular Vesicles Activate WNT Signaling Pathway to Accelerate Intestinal Injury Repair and Reverse Aging**

Lingyan Yang^1,2,3^, Xu Wang^1^, Xiyang Wei^1^, Pei Yu^1^, Shixiang Wang^1^, Yuefang Lin^1^, Yue Yang^4^, Ting Jiang^1^, Yue Liu^1^, Zhiping Qiao^1^, Jiaxiang Zhang^1^, Shicheng Yu^1^, Ye-Guang Chen^1,5,6^, Yun-Shen Chan^1,2^

**This file includes:**

Figures. S1 to S6

Tables S1 to S2


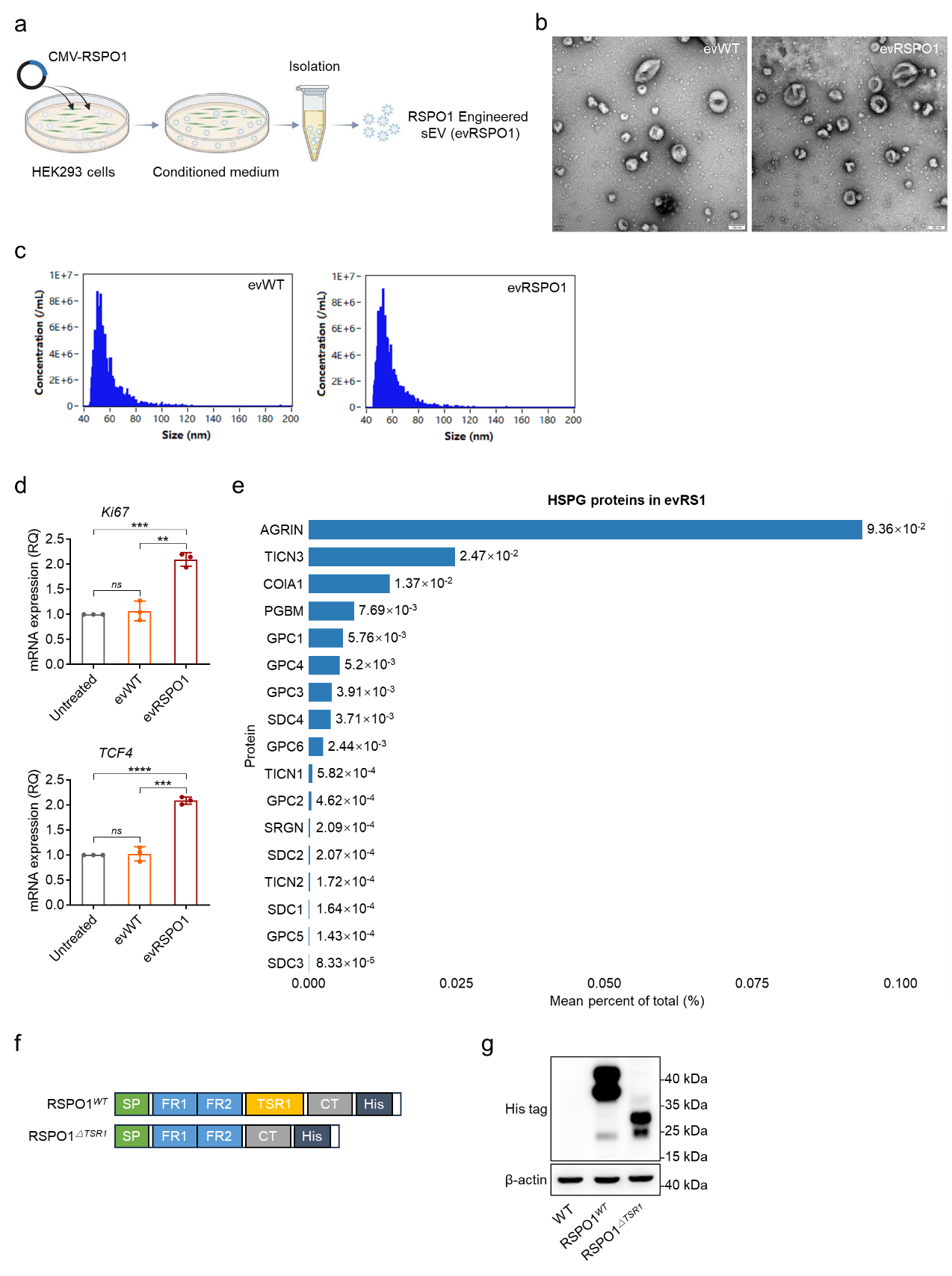


**FIGURE S1** RSPO1 protein engineered sEV isolation, characterization and activity assessment.

1. Schematic illustration of RSPO1 engineered HEK293 cells construction and RSPO1 engineered sEV (evRSPO1) isolation.
2. Transmission electron microscope (TEM) images showing the morphology and size of sEV purified from unmodified cell lines (evWT) and RSPO1-engineered HEK293 cells.
3. NanoFlow analysis showing the concentration and size distribution of evWT and evRSPO1.
4. Real-time PCR analysis of the gene expression level of *Ki67* and *TCF4* in HEK293T cells treated with 1×10^9^ particles/mL evWT or evRSPO1 for 24 h. β-actin was used as a control gene, and the relative fold-change normalized to the untreated group is shown (n=3).
5. LC-MS/MS analysis showing the HSPGs enriched in evRSPO1.
6. Schematic of full-length RSPO1*^WT^* and RSPO1*^ΔTSR1^* overexpression plasmid construction.
7. Western blot analysis of His-tag expression level in RSPO1*^WT^* and RSPO1*^ΔTSR1^* protein overexpression HEK293 cells. β-actin was used as a loading control.


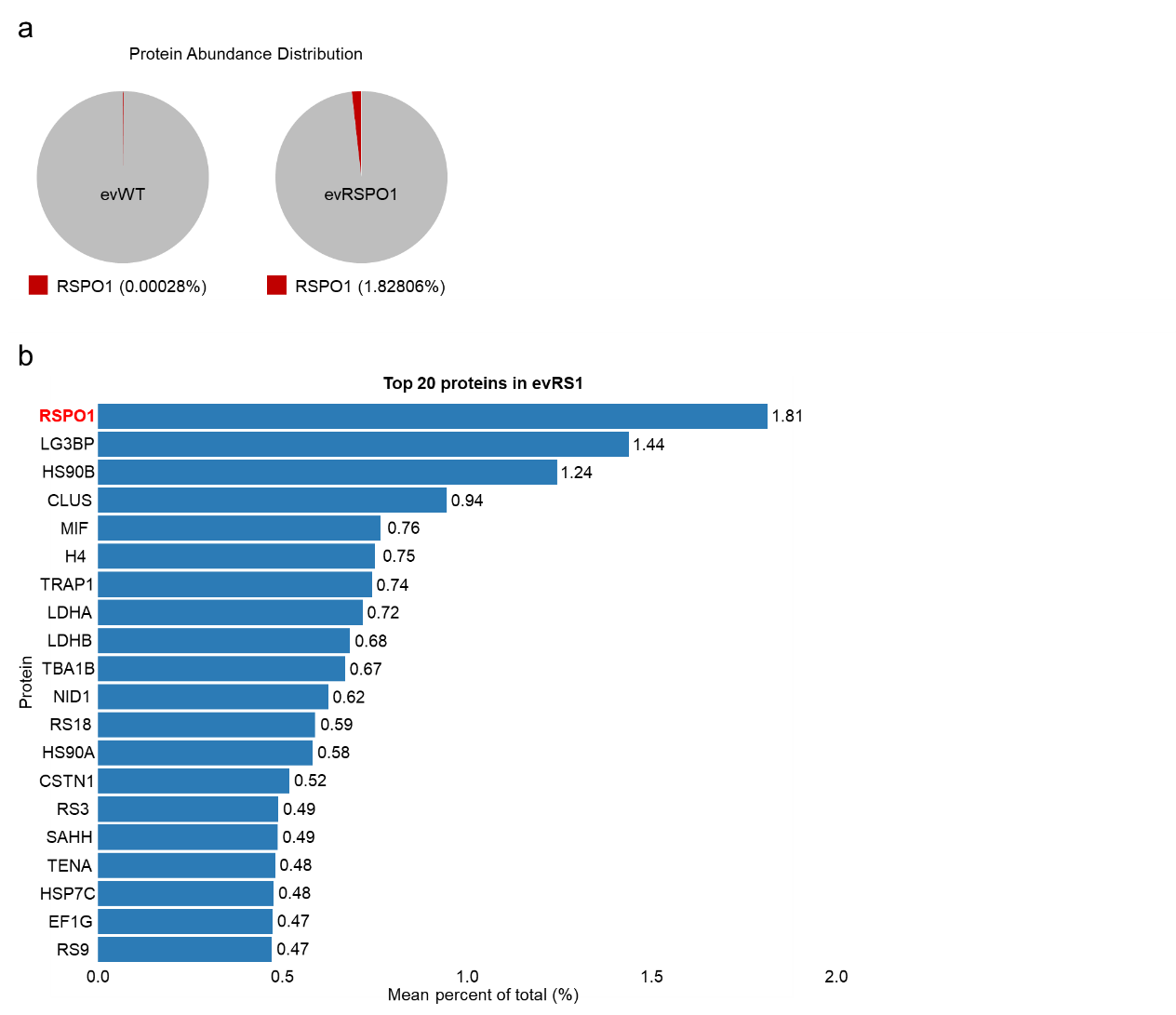


**FIGURE S2** Proportion of RSPO1 protein loading on evRSPO1.

1. Liquid Chromatography Tandem-mass Spectrometry (LC-MS/MS) detected the proportion of RSPO1 in evWT or evRSPO1.
2. LC-MS/MS analysis showing the top 20 proteins enriched in evRSPO1.


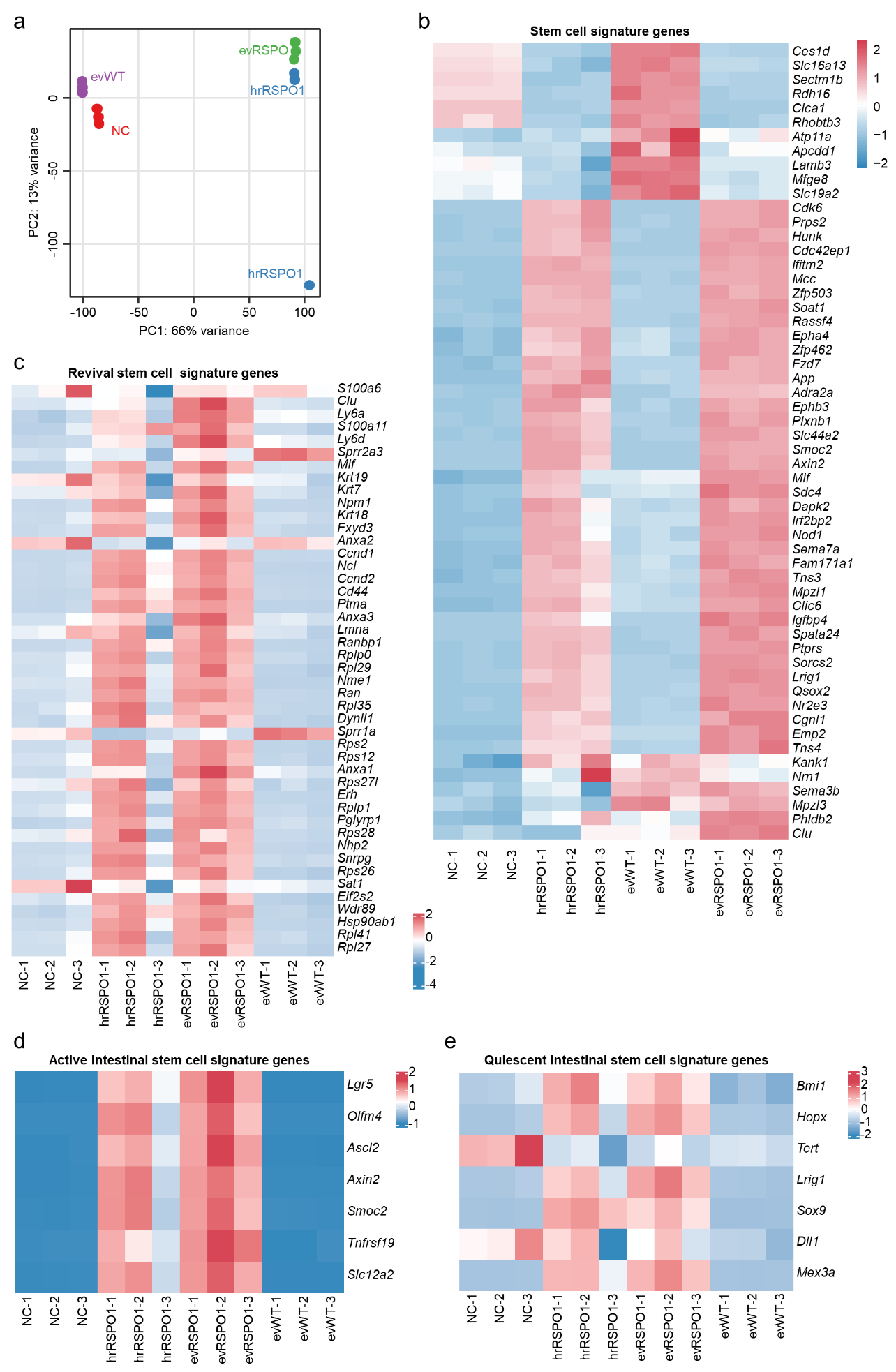


**FIGURE S3** Transcriptomic assay of the evRSPO1 and hrRSPO1 function in the intestinal organoids culture.

1. Principal component analysis (PCA) of gene expression profiles in the intestinal organoids treated with negative control, 100 ng/mL hrRSPO1, 1×10^9^ particles/mL evWT or evRSPO1 for 48 h (n=3).
2. Heatmap showing stem cell signature genes in the intestinal organoids treated with negative control, 100 ng/mL hrRSPO1, 1×10^9^ particles/mL evWT or evRSPO1 for 48 h (n=3).
3. Heatmap showing revival stem cell signature genes in the intestinal organoids treated with negative control, 100 ng/mL hrRSPO1, 1×10^9^ particles/mL evWT or evRSPO1 for 48 h (n=3)
4. Heatmap showing active intestinal stem cell signature genes in the intestinal organoids treated with negative control, 100 ng/mL hrRSPO1, 1×10^9^ particles/mL evWT or evRSPO1 for 48 h (n=3).
5. Heatmap showing quiescent intestinal stem cell signature genes in the intestinal organoids treated with negative control, 100 ng/mL hrRSPO1, 1×10^9^ particles/mL evWT or evRSPO1 for 48 h (n=3).

**
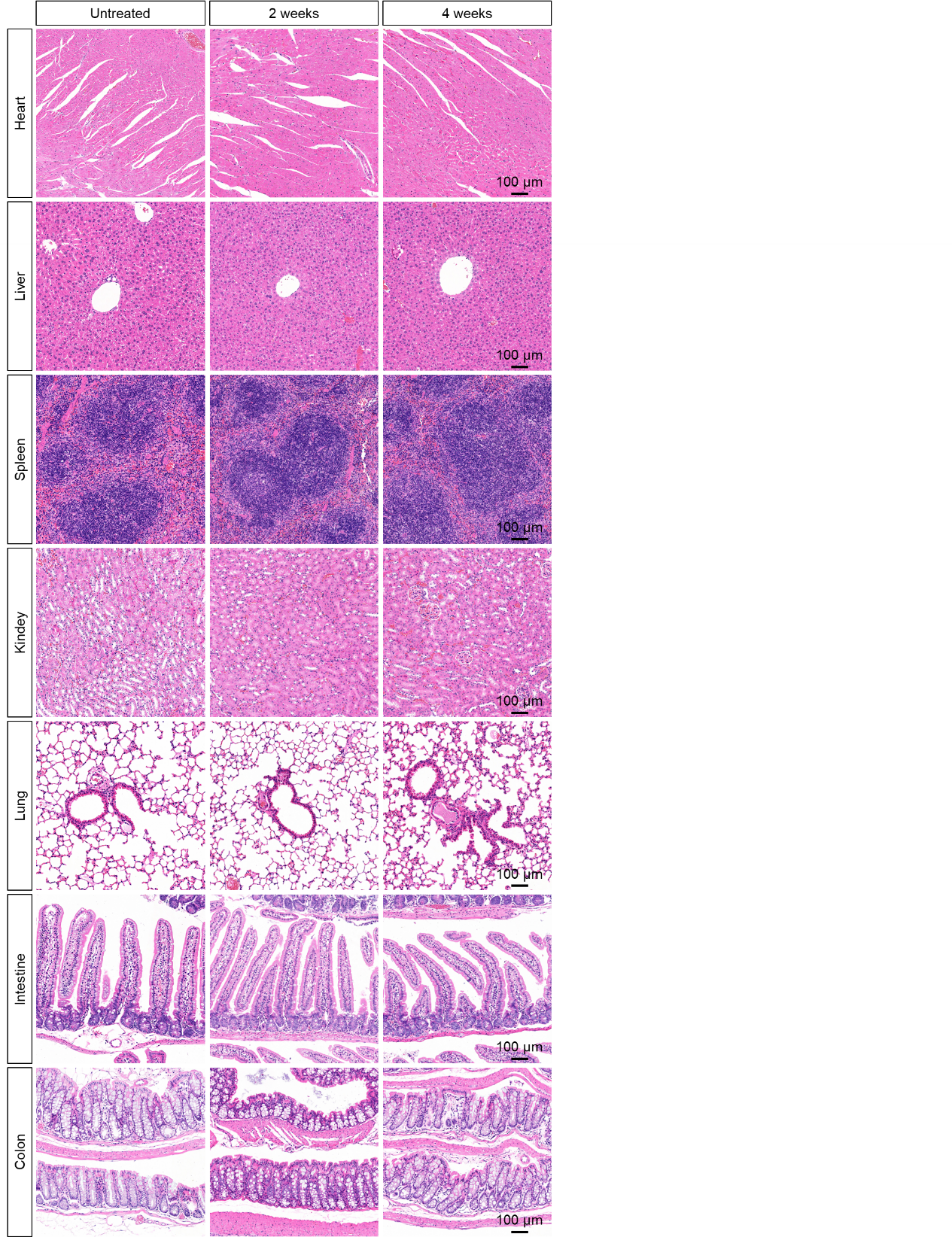
**

**FIGURE S4** Histology of different organs in mice with longer-term evRSPO1 oral administration. Mice were treated with 2 × 10^10^ particles evRSPO1 twice a week for up to 4 weeks.


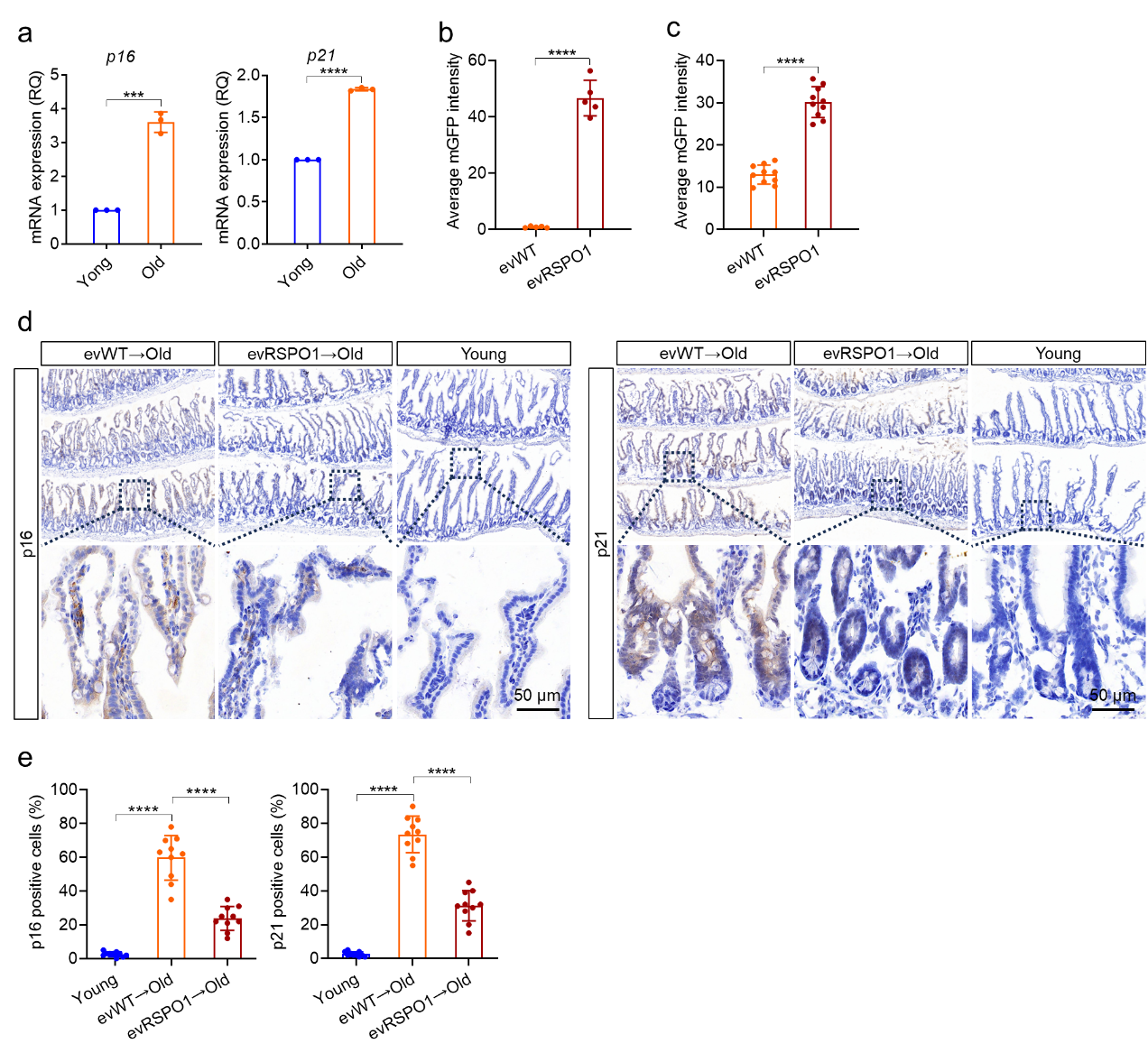


**FIGURE S5** Oral administration of evRSPO1 reversed senescence phenotype in old mouse intestine.

1. Real-time PCR analysis of the expression level of cellular aging marker gene *p16* and *p21* in old mouse-derived intestinal organoids. β-actin was used as a control gene, and the relative fold-change normalized to the untreated group is shown (n=3).
2. Quantification of the Axin2-mGFP signals in the Figure 7a (n = 5).
3. Quantification of the Axin2-mGFP signals in the Figure 7b (n = 10).
4. Immunohistochemistry staining showing the p16 and p21 in intestinal tissue from young mice and old mice after 2 × 10^10^ particles evWT or evRSPO1 oral administration.
5. Quantification the percentage of the p16 and p21 in intestinal tissue from young mice and old mice after 2 × 10^10^ particles evWT or evRSPO1 oral administration (n = 10).

**
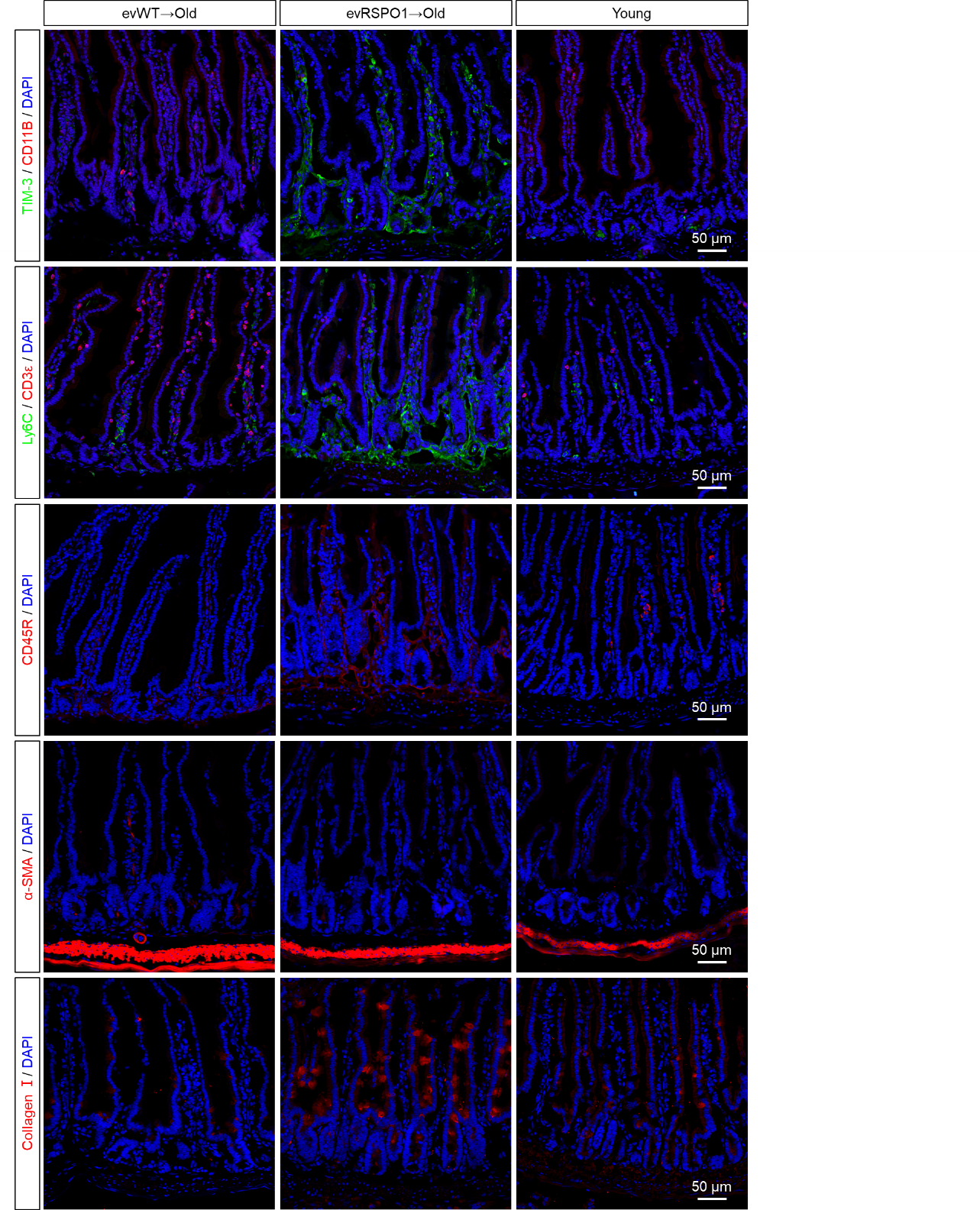
**

**FIGURE S6** Immunofluorescent staining of immune cell and fibroblast cell markers in the intestinal tissue from young mice and old mice after 2 × 10^10^ particles evWT or evRSPO1 oral administration.

Table S1.

Key resources list

| **Antibodies** | **Source** | **Identifier** |
| --- | --- | --- |
| Ms anti-β-actin | Cell signal | Cat. 3700S |
| Ms anti-R-Spondin1 | R&D systems | Cat. MAB4658 |
| Rb anti-Non-phospho (Active) β-Catenin (Ser45) | Cell signal | Cat. 19807S |
| Rb anti-β-Catenin | Cell signal | Cat. 8480S |
| Rb anti-TSG101 | Abcam | Cat. ab125011 |
| Rb Anti-CD63 | Abcam | Cat. ab134045 |
| Rb Anti-CD9 | Abcam | Cat. ab236630 |
| Rb anti-Calnexin | Abcam | Cat. ab133615 |
| Rb anti-HSP70 | Abcam | Cat. ab181606 |
| Rb anti-Ki67 | Abcam | Cat. ab15580 |
| Ms anti-E-cadherin | BD Biosciences | Cat. 610182 |
| Ms anti-IFNγ | Santa Cruz | Cat. Sc-8423 |
| Rb anti-CD45 | Abcam | Cat. ab10558 |
| Ms anti-ZO-1 | Invitrogen | Cat. XL361204 |
| Rb anti-Occluding | Abcam | Cat. ab216327 |
| Rb anti-Olfm4 | Cell signal | Cat. 39141S |
| Rb anti-Muc2 | Abcam | Cat. ab272692 |
| Rb anti-α-SMA | Abmart | Cat. T55295S |
| Rb anti-Collagen I | Abmart | Cat. P28372-B2S |
| Rb anti-CD11B | Abmart | Cat. T55019S |
| Rb anti-Ly6C | Abmart | Cat. MA8959S |
| Rb anti-TIM-3 | Abmart | Cat. MA8957S |
| Ms anti-CD45R | Abmart | Cat. MT13935S |
| Ms anti-CD3ε | Abmart | Cat. TD6594S |
| Goat anti-Rabbit IgG (H+L) Cross-Adsorbed Secondary Antibody, Alexa Fluor™ 488 | Invitrogen | Cat. A-11008 |
| Goat anti-Rabbit IgG (H+L) Cross-Adsorbed Secondary Antibody, Alexa Fluor™ 594 | Invitrogen | Cat. A-11012 |
| Goat anti-Mouse IgG (H+L) Cross-Adsorbed Secondary Antibody, Alexa Fluor™ 594 | Invitrogen | Cat. A-11005 |
| HRP-conjugated Goat anti-Rabbit antibody | Beyotime | Cat. A0208 |
| HRP-conjugated Goat anti-Mouse antibody | Beyotime | Cat. A0216 |

Table S2.

Primers used in this study.

| Gene name |  | Sequence |
| --- | --- | --- |
| *Human AXIN2* | F | GAT CAC TGG CTC CGC GAG |
|  | R | GAA ATC CAT CGC TCT GAG GGG |
| *Human CCND1* | F | AGC TGT GCA TCT ACA CCG AC |
|  | R | GAA ATC GTG CGG GGT CAT TG |
| *Human Ki67* | F | ATCAAAAGGAGCGGGGTCG |
|  | R | ACAACAGGAAGCTGGATACGG |
| *Human TCF4* | F | TCTGAATCCGAAAGCTGCGT |
|  | R | GTCTCCCATTCCAGGGTGTG |
| *Human ACTB* | F | CAA AGT TCA CAA TGT GGC CG |
|  | R | GAC TTC CTG TAA CAA CGC ATC TC |
| *Mouse Lgr5* | F | CCTACTCGAAGACTTACCCAGT |
|  | R | GCATTGGGGTGAATGATAGCA |
| *Mouse Axin2* | F | AACCTATGCCCGTTTCCTCTA |
|  | R | GAGTGTAAAGACTTGGTCCACC |
| *Mouse Ki67* | F | ATCATTGACCGCTCCTTTAGGT |
|  | R | GCTCGCCTTGATGGTTCCT |
| *Mouse Myc* | F | CCCTATTTCATCTGCGACGAG |
|  | R | GAGAAGGACGTAGCGACCG |
| *Mouse Ccnd1* | F | GCGTACCCTGACACCAATCTC |
|  | R | CTCCTCTTCGCACTTCTGCTC |
| *Mouse Pcna* | F | TTTGAGGCACGCCTGATCC |
|  | R | GGAGACGTGAGACGAGTCCAT |
| *Mouse Olfm4* | F | CAGCCACTTTCCAATTTCACTG |
|  | R | GCTGGACATACTCCTTCACCTTA |
| *Mouse Ascl2* | F | AAGCACACCTTGACTGGTACG |
|  | R | AAGTGGACGTTTGCACCTTCA |
| *Mouse Muc2* | F | AGGGCTCGGAACTCCAGAAA |
|  | R | CCAGGGAATCGGTAGACATCG |
| *Mouse Lyz1* | F | GAGACCGAAGCACCGACTATG |
|  | R | CGGTTTTGACATTGTGTTCGC |
| *Mouse p16* | F | CGCAGGTTCTTGGTCACTGT |
|  | R | TGTTCACGAAAGCCAGAGCG |
| *Mouse p21* | F | CCTGGTGATGTCCGACCTG |
|  | R | CCATGAGCGCATCGCAATC |
| *Mouse ACTB* | F | GGCTGTATTCCCCTCCATCG |
|  | R | CCAGTTGGTAACAATGCCATGT |
